# FERM domain–containing protein 6 identifies a subpopulation of varicose nerve fibers in different vertebrate species

**DOI:** 10.1007/s00441-020-03189-7

**Published:** 2020-03-21

**Authors:** Josefa Beck, Michael Kressel

**Affiliations:** Institute of Anatomy and Cell Biology, University of Erlangen, Krankenhausstr. 9, 91054 Erlangen, Germany

**Keywords:** FRMD6, Willin, FERM domain, Expanded, Alzheimer’s disease

## Abstract

FERM domain–containing protein 6 (FRMD6) is a member of the FERM protein superfamily, which is evolutionary highly conserved and has recently been identified as an upstream regulator of the conserved growth-promoting Hippo signaling pathway. In clinical studies, the FRMD6 gene is correlated with high significance to Alzheimer’s disease and cognitive impairment implicating a wider role of this protein in the nervous system. Scare data are available on the localization of endogenous FRMD6 in neural tissues. Using a FRMD6-directed antiserum, we detected specific immunoreactivity in varicose nerve fibers in the rat central and peripheral nervous system. FRMD6-immunoreactive (-ir) neurons were found in the sensory ganglia of cranial nerves, which were marked by a pool of labeled cytoplasmic granules. Cross-species comparative studies detected a morphologically identical fiber population and a comparable fiber distribution in tissues from xenopus and human cranial nerves and ganglia. In the spinal cord, FRMD6-ir was detectable in the terminal endings of primary afferent neurons containing substance P (SP). In the rat diencephalon, FRMD6-ir was co-localized with either SP- or arginine vasopressin–positive fibers in Broca’s diagonal band and the lateral septum. Dense fiber terminals containing both FRMD6-ir and growth hormone-releasing hormone were found in the median eminence. The intimate association of FRMD6 with secretory vesicles was investigated in vitro. Induction of exocytotic vesicles in cultured cells by ectopic expression of the SP precursor molecule preprotachykinin A led to a redistribution and co-localization of endogenous FRMD6 with secretory granules closely mimicking the observations in tissues.

**Electronic supplementary material:**

The online version of this article (10.1007/s00441-020-03189-7) contains supplementary material, which is available to authorized users.

## Introduction

The transition from uni- to multicellular life entailed a new range of cellular tasks for coordinated development, morphogenesis and tissue organization, e.g., planar and apical basal cell polarity, cell proliferation and organ size control, cellular recognition, adhesion and movement. In the context of these demands, the FERM domain containing the superfamily of proteins in animals expanded, which originated at the transition from uni- to multicellular life and is not present in bacteria and plants. The Drosophila genome contains 22 proteins encoding FERM proteins, which expanded to approximately 50 in human (Tepass 2009; Michie et al. 2019).

The defining feature for the FERM domain containing the family of proteins is the presence of a FERM domain, typically at the N-terminus of the protein (with a few exceptions, where the FERM domain is disposed at the C-terminus) and a core alpha helical domain linked to a C-terminal domain, which often bears an F-actin binding site (Diakowski et al. 2006). The presence of a FERM domain enables plasma or endosomal membrane association of the respective protein, either by direct binding to negatively charged phospholipids by virtue of a basic cleft between the F1 and F3 FERM subdomains, or by providing binding sites to a wide array of transmembrane or plasma membrane–associated proteins. The activity of FERM domains is tightly regulated by a conformational switch mechanism, whereby the C-terminal domain wraps around the FERM domain masking its binding sites. Phosphorylation of key residues and lipid binding are required for activation of the auto-inhibited dormant molecule and lead to unfolding and unmasking of its binding sites (Bretscher et al. 2002). FERM proteins are characteristically concentrated at structural features of the plasma membrane, i.e., microvilli, stereocilia, filopodia, podocytes, or at endomembrane remodeling sites involving, e.g., phagocytosis, endosome recycling, vesicular trafficking and maturation. FERM domain proteins, therefore, play a key role in cell adhesion, migration, polarity and vesicular trafficking (Kvalvaag et al. 2013; Muriel et al. 2015). Tissue- and development-specific alternative splicing adds to the functional diversity of the FERM protein family, which modulates subcellular localization, autophosphorylation and binding partner interactions (Schmucker et al. 1999; Debrand et al. 2008; Armendáriz et al. 2014; Baines et al. 2014).

Recently, a new member of the FERM family has been characterized as a binding partner to neurofascin, which has been called willin or FERM domain–containing protein 6 (FRMD6). Willin is a 622 amino acid protein with a predicted molecular weight of 71 kDa and is widely expressed in both neural and non-neural tissues (Gunn-Moore et al. 2005; Moleirinho et al. 2013a). In the peripheral nervous system, willin mRNA was predominantly observed in the perineurium and in endoneural fibroblasts by in situ hybridization of sciatic nerves (Moleirinho et al. 2013b). Expression of GFP-tagged willin in cultured cell lines is associated with punctate staining throughout the cytoplasm, the plasma membrane, the cell nucleus and the cell-cell junctions (Gunn-Moore et al. 2005; Fanbo et al. 2015). Willin is recruited to cell-cell junctions by nectins (Ishiuchi and Takeichi 2012) and regulates by binding to aPKC the size of the apical plasma membrane domain (Ishiuchi and Takeichi 2011). Immunohistochemical studies have described the intracellular distribution of endogenous FRMD6 at the plasma membrane and diffusely in the cytoplasm and cell nucleus of oral squamous cells (Madan et al. 2006). Mechanisms that influence the distribution of FRMD6 need yet to be further investigated (Gunn-Moore et al. 2005).

We now describe, using an antiserum raised against rat FRMD6, that this protein immunohistochemically is associated with secretory vesicles and varicose nerve fibers of specific neuronal subsystems in the central and peripheral nervous system.

## Materials and methods

### Animals and tissue preparation

For all procedures performed upon animals, the federal animal welfare legislation implemented by the local government and approved by the University of Erlangen was followed. For this study, one C57/Bl6 mouse and 27 adult Wistar rats (200–400 g) of either sex were used. The animals were euthanized with a single intraperitoneal overdose of thiopental (Trapanal, Inresa, Germany) (250 mg/kg) and, when fully unresponsive, were perfused through the ascending aorta initially with saline containing 10 IU heparin/ml until the effluent out of the right atrial appendage became clear, immediately followed by 300 ml of 3% paraformaldehyde in 0.1 M PO_4_ buffer, pH 7.2. Alternatively, in five rats, buffered picric-formaldehyde solution (0.1 M PO_4_, 3% paraformaldehyde, 16% saturated picric acid solution) was used as a fixative. For this study, the cranial nerves II–XII together with the adjacent sensory ganglia of the fifth, ninth and tenth cranial nerves and a section of the spinal cord were dissected out. In four rats, the brain was removed. Additionally, cranial nerves, brains, spinal cords and lung tissues from five *Xenopus laevis* African clawed frogs were preserved. For this, the animals were transferred to a container containing 0.2% MS222 until all reflexes to stimuli became extinct and they were then perfusion-fixed with 3% paraformaldehyde in 0.1 M PO_4_ buffer, pH 7.2 through the ascending aorta, as described above. The cranial nerves II–XII and the trigeminal ganglia from two human corpses (72 and 87 years old), who had donated their bodies to the Anatomy Department, were dissected 12–15h postmortem and fixed by immersion in 4% PFA in PO_4_ buffer. The spinal cords of two rainbow trouts, obtained from a local fish dealer, were removed immediately after killing and immersion-fixed in phosphate-buffered 4% formalin solution. A third spinal cord was dissected out from a trout, which was perfusion-fixed 30 min after death with 3% paraformaldehyde as described above. After fixation, all tissues were stored overnight at 4 °C in 0.1 M PO_4_ buffer, transferred to 0.1 M PO_4_ buffer containing 15% sucrose for 24 h, rapidly frozen in isopentane at − 75 °C and stored at − 20 °C until preparation of cryosections.

### Antibodies and immunohistochemistry

Fourteen- to eighteen-μm-thick cryostat sections were air dried onto glass slides; rehydrated in TBS buffer (0.1 M Tris-HCl, pH 7.5, 0.15 M NaCl, 0.05% Tween 20); preincubated for 1 h in TBS containing 1% bovine serum albumin (BSA), 0.5% Triton X 100 and 5% normal donkey serum; and incubated with the primary antibody overnight in the same buffer. As primary antibody, we used a rabbit anti-FRMD 6 antiserum (Santa Cruz Biotechnology), which had been raised against an internal region in the N-terminal half of rat FRMD6, in a 1:1000 dilution. For detection of FRMD6 in Western blots, a FRMD6 antiserum directed against a different epitope in the C-terminus of the protein (aa 446-580) (Sigma-Aldrich) was used. For a complete list of all antibodies, see ESM, Table S1. Secondary antibodies were all from Jackson Immuno Research Laboratories, which had been cross-absorbed with multiple species and were used in a dilution of 1:2000. After incubation with the secondary antibodies, the slides were mounted in TBS-Glycerin (pH 8.6). For the preabsorption control, the primary antibody was incubated with the corresponding peptide (sc-138006P), to which the antibody has been raised, in a 1:10 ratio by weight prior to its application to the cryoslides. The slides were examined with a Nikon C1 confocal laser-scanning microscope. Apart from adjustment of contrast and brightness, no secondary image processing was done.

### Immunoprecipitation and Western blotting

MCF-7 human mammary adenocarcinoma (ECACC 86012803), NIH3T3 mouse embryo fibroblast (ECACC 93061524) and AtT-20/D16v-F2 mouse pituitary tumor cells (ECACC 94050406) were obtained from the European Collection of Authenticated Cell Cultures and were cultured under standard cell culture conditions at 37 °C and 5% CO_2_, detached from the culture flask by digestion with Accutase solution (Sigma-Aldrich) and pelleted. Cell and crude synaptosomal fractions (P2 fraction) of rat and xenopus brains were prepared according to the protocol of Hell and Jahn (2006). Cell and synaptosomal pellets were dissolved in Laemmli sample buffer and resolved on 4–12% SDS-polyacrylamide gels and transferred to polyvinylidene difluoride (PVDF) membranes.

For immunoprecipitation, semi-confluent cell cultures from a 75-cm^2^ flask were detached, washed in phosphate-buffered saline, lysed in 7 ml CelLytic M (Sigma-Aldrich), supplemented with one tablet complete ULTRA protease inhibitor cocktail (Roche) and 0.04 u/μl benzonase nuclease and incubated at 4 °C for 1 h. Rat spinal cord sections were shock-frozen and grinded in liquid nitrogen and dissolved in 7 ml buffer. After a brief centrifugation at 1000*g* for 5 min, 50 μl Protein G–coated paramagnetic beads (Life Technologies) preincubated with 2 μg of the N-terminus-specific FRMD6 antiserum and subsequently cross-linked with bis(sulfosuccinimidyl)suberate sodium salt (BS3) was added to the supernatant. After incubation for 40 min, the beads were thoroughly washed and eluted in sample buffer under reducing conditions. Samples were separated on a 4–12% Bis-Tris PAGE under reducing conditions and blotted onto PVDF membranes. Blots were incubated with an anti-FRMD6 antiserum raised against the C-terminus of the protein and as secondary antibody, a biotinylated mouse anti-rabbit monoclonal antibody (γ-chain-specific, Clone RG-96) (Sigma-Aldrich) was used. Bands were visualized by streptavidin peroxidase incubation and chemiluminescence detection.

### Cloning, cell culture and transfection

Human preprotachykinin A (gene ID: 6863) containing the signal peptide was amplified by RT-PCR using a human brain cDNA and agGAATTCATGAAAATCCTCGTGGCCTTGGCAGTC and aaGGATCCCCACGTCTTCTTTCATAATTCTGCATTGCACTCC as forward and reverse primers, respectively (gene-specific sequences underlined) and cloned into the pFusionRed-N (Evrogen) eukaryotic expression plasmid by the EcoRI and BamHI restriction sites.

For transfection experiments, MCF-7 and AtT-20 cells were seeded onto Nunc chamber slides (Nunc, Naperville, IL, 60566, USA) coated with fibronectin or laminin and allowed to attach for 4 h. Universal transfection reagent obtained from Sigma-Aldrich was used according to the manufacturer instructions. For expression, 1 μg purified plasmid was added per well. Twenty-four hours after transfection, cells were fixed with 3% paraformaldehyde in phosphate-buffered saline and processed for immunofluorescence.

## Results

### Sensitivity, specificity and epitope mapping of the FRMD6 antibody

**Fig. 1:**
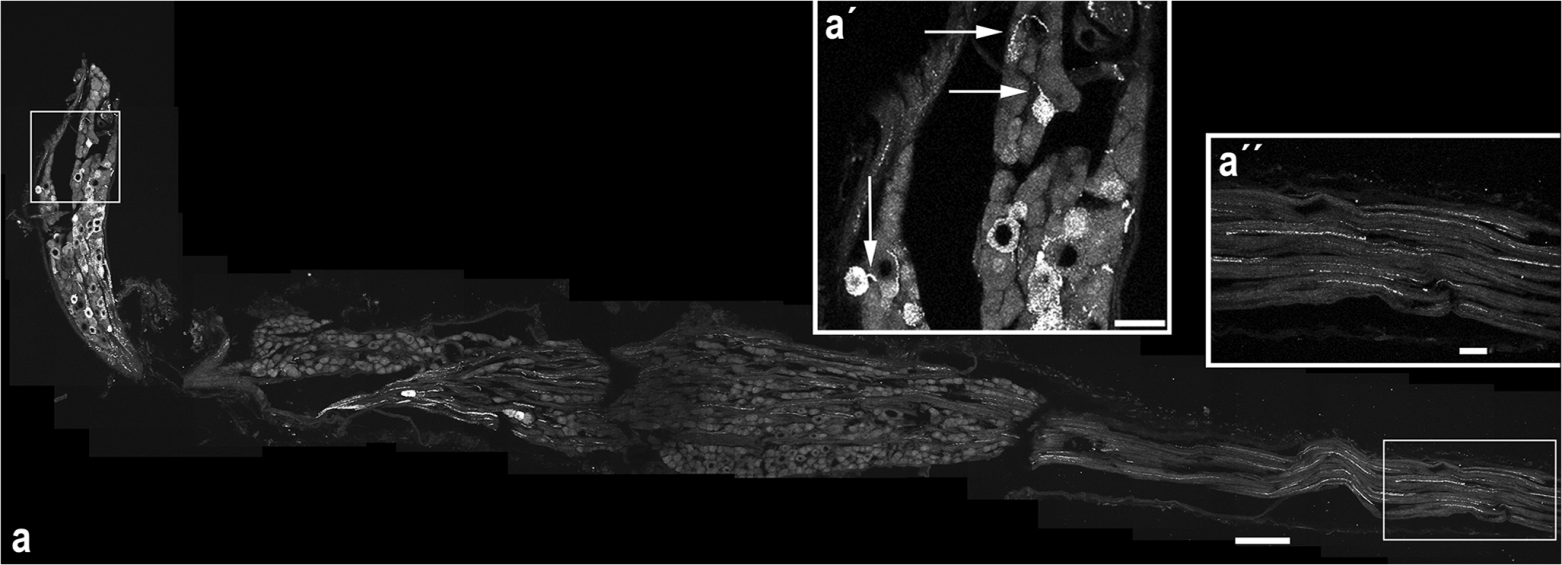
FRMD6-ir neuronal cell bodies and varicose fibers. **a** The image displays a section of the rat vagus nerve cut immediately below the cranial base including the jugular and nodose vagal ganglia stained by the FRMD6 antiserum. Confocal stacks from nine overlapping images were z-projected and the sub-images stitched together by the ImageJ stitching plugin to a composite image. The jugular ganglion is to be seen in a vertical position on the left, the nodose ganglion represents the enlarged horizontally lying bulb in the middle tapering towards the cervical vagus nerve to the right side of the image. Individually stained neuronal cell bodies can be observed exclusively in the jugular ganglion, while the nodose ganglion shows only fibers of passage but no fluorescent cell bodies. Inset (a′) shows a magnification of the boxed area across the jugular ganglion. Neuronal cell bodies are marked by fluorescent cytoplasmic granules, which occasionally can be observed on the transport route to the axon (arrows). Inset (a″) shows a magnification of the boxed area across the cervical vagus nerve. Individual FRMD6-ir varicose nerve fibers can be observed in the vagus trunk. Scale bar 200 μm (**a**), 50 μm (a′, a″)

FRMD6 immunohistochemistry revealed intensely fluorescent small varicose nerve fibers in the rat peripheral and central nervous system. Additionally, in trigeminal, jugular and petrose ganglia, neuronal FRMD6-ir cell bodies were outlined by an accumulation of distinctly fluorescent cytoplasmic vesicles, which in some instances could be observed on their transport route from the cytoplasm into the axon (Fig. 1). The antiserum was used in a dilution of 1:1000. In still higher dilutions of up to 1:5000, specific labeling was still detectable but reduced in signal intensity. Preabsorption tests were implemented using different animal species and different tissues. The labeling was specific, as after preabsorption of the antibody with the corresponding peptide, no residual fluorescence, i.e., no labeled nerve fibers nor cytoplasmic staining, could be observed in tissues of rat as well as of xenopus origin (ESM, Fig. S1). As has been observed by numerous investigators with substance P (SP) immunohistochemistry of rat tissues, which labels a comparable small varicose nerve fiber population, the intensity of the immunoreaction varied widely in rats from animal to animal (Ljungdahl et al. 1978). Younger rats (200–250 g body weight) tended to have more numerous and more intensely stained fibers. No obvious interindividual variability in staining intensity comparable with the rat was noted in tissues derived from xenopus or fish. Tissues fixed with buffered picric-formaldehyde solution had slightly more intensely labeled fibers compared with tissues from control littermates fixed with paraformaldehyde alone but irrespective of staining intensity, no further differences could be observed between individual animals and different fixation recipes.

**Fig. 2:**
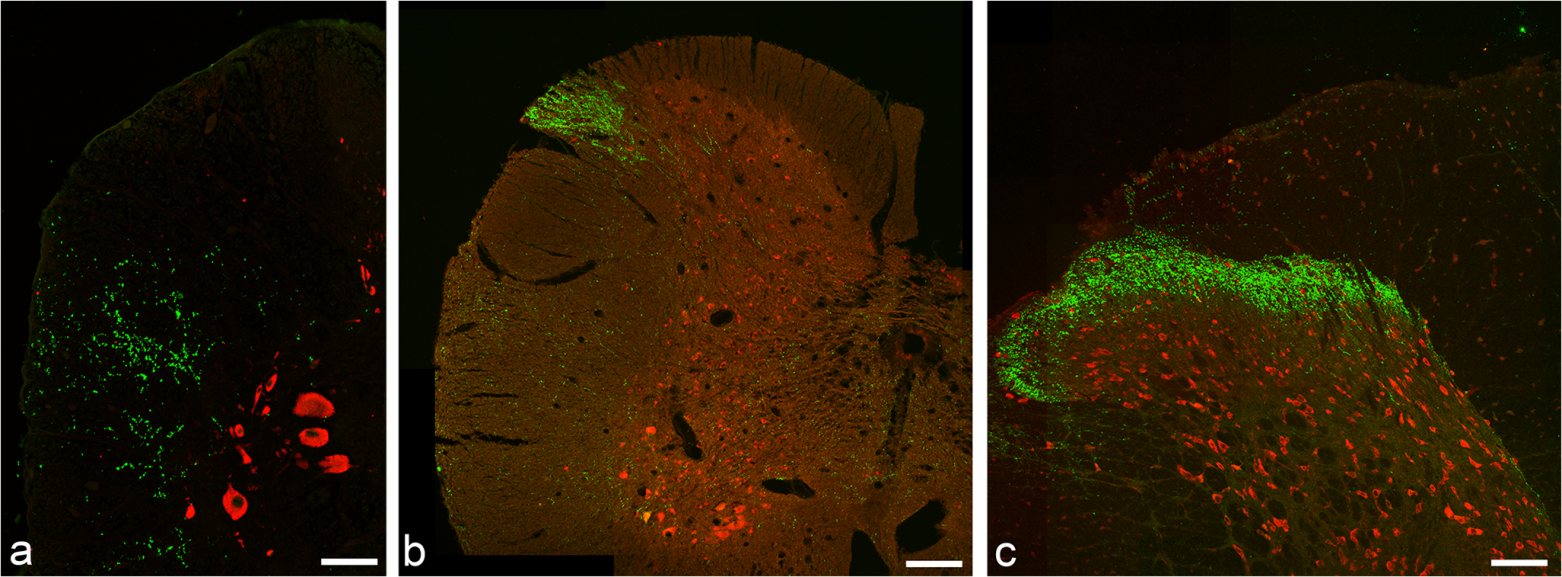
Spinal cord sections from rainbow trout (**a**), xenopus (**b**) and rat (**c**), respectively, were double stained for FRMD6 (green) and HuC (red), which is a marker for neuronal perikarya. Single FRMD6-ir fibers and terminal endings are displayed in the dorsolateral area of the trout spinal cord (**a**). A dense network of terminal afferent endings in lamina II of the rat dorsal horn is highlighted by FRMD6 (**c**) and in the Lissauer tract of the xenopus spinal cord (**b**). Scale bar 100 μm

FRMD6 is an evolutionary highly conserved protein. The N-terminal half (aa 1-307), of the protein to which the antibody has been raised, displays 84% conserved residues across the four species *Danio rerio*, xenopus, rat and humans. The antiserum, although raised against FRMD6 of rat origin, could, therefore, be expected to cross-react with multiple species. Spinal cords from rainbow trout, xenopus, mice and rat and the trigeminal ganglia from two human body donors were obtained and immunostained with the FRMD6 antibody. In all tissues examined, the antiserum exclusively labeled neurons and varicose nerve fibers, which morphologically appeared to belong to an identical fiber population (Fig. 2; ESM, Fig. S2).

As expression of FRMD6 has been reported in a wide range of tissues and cells, MCF-7 and AtT-20 cell lines were additionally probed with the antiserum. In MCF-7 cells, the antiserum highlighted cell-cell junctions with only a punctate and diffuse cytoplasmic fluorescence. In AtT-20 cells, a pool of larger and smaller cytoplasmic granules was observed while cell boundaries were indistinct. After preabsorption of the antiserum, the immunoreactivity in AtT-20 cells vanished, except for a fraction of the smaller vesicles, which still persisted and represented, therefore, unspecific background staining (ESM, Fig. S1). It was noted that the overall staining intensity was not uniform but varied to some extent between cell lines and passages. The specific reasons for this could not be elucidated. Overall, younger and exponentially growing cells tended to display more distinct staining compared with higher and more confluent passages.

**Fig. 3:**
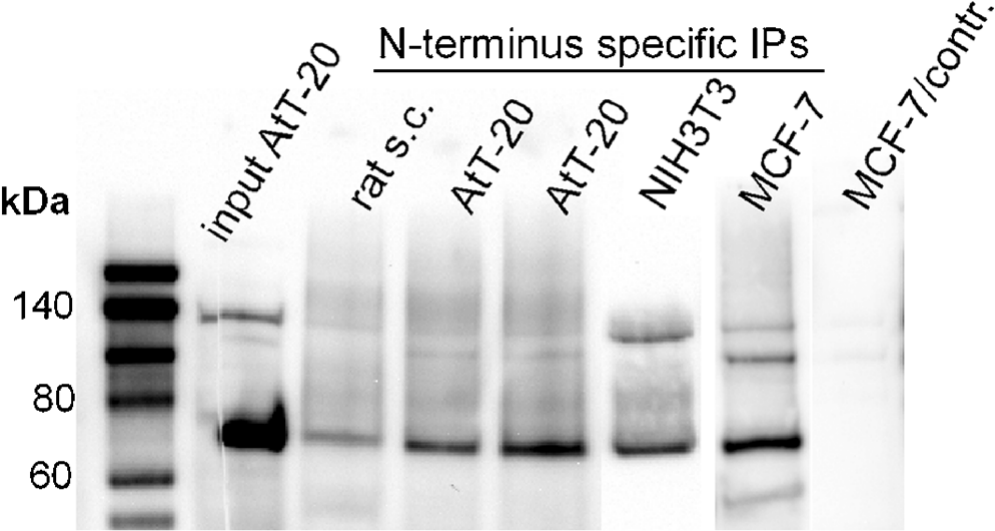
Lane 1: biotinylated molecular weight marker. Lane 2: SDS extract of AtT-20 whole cell lysate probed with the C-terminus-specific antibody. To show the specificity of the antibody, lane 2 was cut into two halves prior to incubation with the primary antibody (cutting line indicated by a punctate vertical line) and both parts were incubated in separate trays with different antisera. The left side of lane 2 was incubated as control with an antiserum against green fluorescent protein, while the right part of lane 2 was incubated with the FRMD6 C-terminus-specific antiserum. Both parts of lane 2 were finally put together again before chemiluminescence imaging. A single specific band at 70 kDa is apparent solely on the right part of lane 2 exposed to the FRMD6 C-terminus-specific antibody, while the left part shows no signal at the corresponding molecular weight. Lanes 3–7: FRMD6 immunoprecipitation in different tissues with the FRMD6 N-terminus-specific antiserum. Immunoprecipitates (IPs) were probed with the FRMD6 C-terminus-specific antibody as in lane 2. Lane 3: a homogenate of rat spinal cord displays no significant band. Lanes 4 and 5: homogenates from two different AtT-20 cell cultures with identical cell densities. A specific band at 70 kDa but with variable intensity is apparent. Lanes 6 and 7: NIH3T3 and MCF-7 cultures, respectively. Lane 8: a control is shown performed under identical conditions with a parallel MCF-7 culture, except that for immunoprecipitation, a rabbit antiserum raised against an unrelated protein (human SGSM3) was used. The entire blotting membrane area is displayed in ESM, Fig. S3a

Sodium dodecyl sulfate (SDS) extracts of homogenized rat brain tissues, as well as of cultured cell lines, were subjected to polyacrylamide gel electrophoresis, subsequent Western blotting and probing with the FRMD6 antiserum. However, exposure to SDS allowed the denaturation of the epitope detected by the FRMD6 antiserum, which precluded its use in probing Western blots. In contrast, a FRMD6 antiserum raised against a different epitope in the C-terminus of the protein (aa 446-580) reliably stained a specific band of about 70 kDa at the expected molecular weight of the protein, as already described by Xu et al. (2016) in FRMD6 shRNA experiments. This band was consistently found in crude and synaptosomal extracts of xenopus and rat brains, as well as in all cultured cell lines. This enabled us to perform immunoprecipitation with the N-terminal FRMD6 antibody and to probe the immunoprecipitated proteins after SDS gel electrophoresis and Western blotting with the second FRMD6 C-terminus-specific antibody. The N-terminus-specific antibody precipitated a specific band, as detected by the C-terminus-specific antibody, corresponding to the full-length FRMD6 protein at 70 kDa in protein extracts of the cell lines AtT-20, NIH3T3 and MCF-7 (Fig. 3). Additionally, an unspecific band at 140 kDa was co-precipitated. No distinct signal was detected in extracts of rat spinal cord. As a control, an immunoprecipitation under identical conditions with a rabbit antiserum raised against an unrelated protein was performed, which displayed no specific band at 70 kDa.

Despite its specificity in immunoblots, the C-terminus-specific antibody showed in our hands rather diffuse but no distinct immunofluorescence in formaldehyde-fixed tissues, which could, because of unavailability of the respective antigen, not be verified by preabsorption controls. The C-terminus-specific antiserum was, therefore, omitted from further immunofluorescence analysis, although there are reports of its use in immunohistochemistry (De Sousa E Melo et al. 2013).

According to the manufacturer, the FRMD6 antiserum has been raised against the N-terminal half of FRMD6 of rat origin. We could track down the epitope detected by the antiserum to the human exon 6/7 boundary, as cDNA constructs comprising exons 1–8 created a positive signal in immunofluorescence but not a construct narrowed down to exons 1–6. Consequently, a fusion protein construct of the coding sequence of this region of rat origin (aa 217-240) fused to the C-terminus of enhanced green fluorescent protein (EGFP) could be specifically detected by the FRMD6 antiserum in transfected cells (ESM, Fig. S3b-c). In contrast, no signal was detected for the EGFP moiety alone, as well as for the entire fusion protein construct after preabsorption of the FRMD6 antiserum with the respective antigen.

### FRMD6-positive fiber populations across different species

To find out the specific fiber categories stained by the FRMD6 antiserum, we examined systematically the cranial nerves II–XII and the adjacent trigeminal, jugular and petrose ganglia in three different species, i.e., xenopus, rat and human, taking advantage of the unique fiber composition of each cranial nerve, which should facilitate the allocation of labeled fibers to specific fiber categories. Furthermore, as the Drosophila homologue to FRMD6 is a binding partner of the neurofibromatosis type 2 protein, which upon mutation characteristically leads to bilateral tumors of the eight cranial nerve in the human, we were particularly interested in the comparative fiber distribution of the cranial nerves. FRMD6-positive fibers were absent in nerves III, IV, VI, VII and XII containing exclusively or predominantly motor fibers, as well as in the purely sensory second and eight cranial nerve. A significant FRMD6-positive fiber population was, however, found in the branchial nerves V, IX and X (ESM, Tables S2–4). Labeled fibers were of small caliber and had varicose beads on a string appearance, typically known for peptidergic fibers. This fiber distribution was remarkably uniform across all the three species examined with the highest incidence of positive fibers in the vagal nerve. FRMD6-ir neurons were observed in the corresponding sensory ganglia, i.e., the trigeminal, glossopharyngeal and vagal ganglia (ESM, Fig. S1a, S2). A striking inhomogeneity in the distribution of marked neurons was observed in the vagal ganglia, as FRMD6-ir neurons in the jugular ganglion far outnumbered marked neurons in the nodose ganglion, which contained mainly fibers of passage (Fig. 1; ESM, Table S5). The jugular vs. the nodose ganglion differs in embryological origin, neurochemistry and innervated target structures with the jugular ganglion more similar to a spinal ganglion (Nassenstein et al. 2010). It was concluded that FRMD6 labels in an evolutionary conserved pattern, a small caliber varicose fiber category occurring predominantly in sensory ganglia of the spinal ganglion type.

### FRMD6 and SP colocalize in central and peripheral endings of afferent neurons

**Fig. 4:**
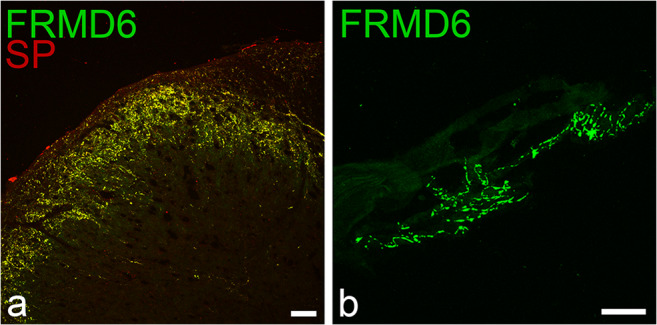
**a** Dorsal horn of the rat spinal cord double-stained for FRMD6 in the green fluorescence channel and SP in the red channel. The overlay image of both channels demonstrates extensive co-localization of SP in FRMD6-ir fibers resulting in the yellow coloration. The individual fluorescence channels are displayed in ESM, Fig. S4c´c´´. **b** Terminal nerve endings of large caliber FRMD6-ir nerve fibers at the lung hilus of xenopus. Bar = 50 μm

To track down the specific varicose fiber subpopulation stained by FRMD6, we performed immunofluorescence staining on rat spinal cord cryosections, where the neurochemical and functional properties of all afferent fibers have been extensively investigated and are very well-known. FRMD6-ir fibers were observed exclusively in the dorsal root and displayed a dense network of terminal endings within lamina I and II of the rat dorsal horn (Fig. 2c). Most of the afferents to the substantia gelatinosa are nociceptors and can be classified into two major subtypes, one containing neuropeptides such as substance P (SP) and calcitonin-gene-related peptide (CGRP), which coexist in secretory vesicles, whereas the other group lacks peptides and binds the plant isolectin I-B4 from *Griffonia simplicifolia* (I-B4) (Stucky and Lewin 1999). Triple immunofluorescence of FRMD6 together with I-B4 and either SP or CGRP in the rat spinal cord clearly showed that FRMD6-ir fibers extensively colocalized with SP or CGRP but not with I-B4 (Fig. 4a). Furthermore, the terminal endings of the FRMD6/SP/CGRP positive fiber population and the I-B4 subtype mostly did not overlap, the first terminating more superficially within lamina II compared with the I-B4 binding subtype (ESM, Fig. S4a-a´´´). This termination pattern of small-diameter afferent fibers in overlapping but distinct sublayers within lamina II has been described before. Peptidergic fibers terminate in the outer lamina II, whereas vanilloid receptor–positive and I-B4 binding fibers terminate in the inner sublayer of lamina II (Guo et al. 1999). In xenopus spinal cord sections, a dense network of FRMD6-labeled varicose terminals was observed in the Lissauer tract below the entrance of the dorsal root, which is homologous to lamina I–II of the mammalian spinal cord (Fig. 2b). Comparable with the rat FRMD6 extensively colocalized with SP, I-B4 binding fibers represented a separate subgroup (ESM, Fig. S4b-b´´´). To probe the extent of colocalization of FRMD6 with SP, the nodose, jugular and petrose ganglia together with the intervening NIX and NX fibers were removed en bloc from a rat animal, cryosectioned and analyzed for colocalization of FRMD6 and SP. All FRMD6-ir fibers and neurons were double-stained for SP, while only 46% of SP-positive neurons were FRMD6-ir (ESM, Table S5). At higher magnification, it became evident that in FRMD6 and SP double-positive neurons, both markers extensively colocalized at cytoplasmic vesicles. Two antisera raised against SP from two different species were employed, i.e., guinea pig and goat. Both gave identical results with the only difference that the antiserum from guinea pig displayed more intense immunofluorescence.

Additionally, we chose to study the peripheral endings of vagal afferent nerves in lung tissues, as we noted in the rat that FRMD6-ir fibers were sparse in the subdiaphragmatic part of the vagus nerve, while most positive fibers branched off in the thoracic part of the vagal nerve. In xenopus, a prominent vagal branch to the lungs could easily be identified, which was examined, therefore, from its entrance to the lung hilar area into the lung tissue proper. Three types of terminal endings in xenopus lung tissues could be identified: (i) large caliber fibers in the region of the lung hilus, which showed characteristic contorted end bulbs similar to the type of endings already described in the frog lung by Smirnow (1888) (Fig. 4b); (ii) small caliber fibers within the muscle bundles of the lung septa and around the walls of the pulmonary blood vessels; (iii) in nerve fibers in the submucosal layer. In all instances, extensive colocalization of SP and FRMD6 was observed (ESM, Fig. S5).

### FRMD6-ir in an anuran brain

An entire xenopus brain from the basis of the olfactory bulbs up to the transition to the spinal cord was cut into 16-μm cryosections and stained for FRMD6 in conjunction with HuC as a counterstain for neuronal cell bodies. Another xenopus brain was cut horizontally into cryosections. FRMD6-ir was found in all parts of the xenopus brain. Rostrally, the internal granule cell layer of the olfactory bulb was permeated by numerous FRMD6-ir fibers. In the forebrain, the ventral striatum and nucleus accumbens were highlighted by a very dense network of FRMD6-ir fiber terminals (ESM, Fig. S6). In the lateral septal area, a moderately dense plexus of FRMD6-positive fibers was observed, while only single fibers were seen in the medial pallium and almost no fibers in the dorsal pallium. In the posterior part of the telencephalon, a high-density fiber plexus was observed lateral to the amygdaloid complex and the entire preoptic region was permeated by many individual fibers. At the level of the optic chiasma, numerous positive fibers were observed throughout the entire neuropil of the diencephalon. The neuropil of the nucleus of Bellonci was highlighted by an intense FRMD6-ir fiber plexus. In the dorsal diencephalon, a dense FRMD6-ir fiber plexus was detected at the boundary to the habenular nuclei, while the nuclei themselves were devoid of labeling. In the thalamic region, a dense fiber network was found in a widespread area in the vicinity and within the central thalamic nucleus and in the neuropil of the thalamopretectal field. The interpeduncular nucleus was highlighted by intense FRMD6-ir. In the optic tectum, numerous positive fibers were detected running perpendicularly to the frontal plane in the stratum opticum in the superficial half of the optic tectum. Beneath, a dense horizontal lamina of FRMD6-ir was detected corresponding to layers VII/VIII of the optic tectum. Comparably, only a sparse innervation by FRMD6-ir fibers was found in the rhombencephalon. In the descending tract of the trigeminal nerve, as well as in rostral continuation of the Lissauer tract, scattered FRMD6-ir fibers were observed but to a lesser extent compared with the Lissuer tract of the spinal cord. Additionally, beneath the floor of the fourth ventricle in the central gray matter between the left and right sulcus limitans, numerous fibers were detected running mostly perpendicular to the frontal plane.

### FRMD6 colocalizes with different neuropeptides in the rat diencephalic region

An immunofluorescence analysis of the entire diencephalic region in the rat brain was undertaken, as neural networks using peptidergic transmission with different neuropeptides are concentrated in this region. Four rat brains were cut into serial frontal sections rostrally from the diagonal band of Broca up to the level of the arcuate nucleus and double or triple immunofluorescence staining of FRMD6 in combination with SP and all peptidergic hypothalamic hypophysiotropic hormones was performed.

**Fig. 5:**
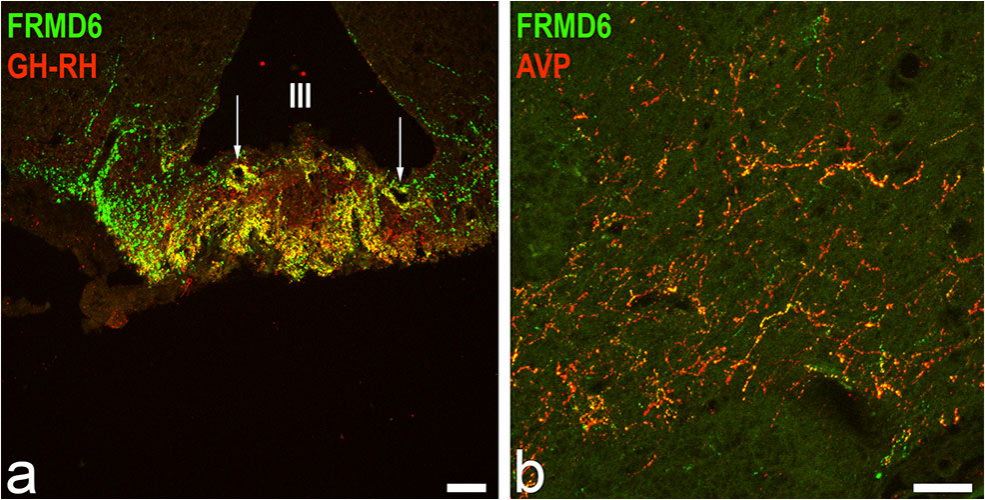
FRMD6-ir in the rat diencephalon. Double immunofluorescence of FRMD6 (green) in conjunction with GH-RH (red) or AVP (red) in the median eminence of the rat hypothalamus (**a**) and in Broca’s diagonal band (**b**), respectively. **a** Intense co-localization (yellow merge color) of FRMD6 and GH-RH is seen in the external layer of the median eminence and in the large capillary loops (arrows) beneath the floor of the third ventricle (III). **b** Extensive co-localization of FRMD6-ir with AVP-ir in nerve fibers with a terminal-like morphology in the vertical limb of the diagonal band of Broca is evident by the yellow coloration of the fibers. The individual images for each fluorescence channel of Fig. 5 are shown in ESM, Fig. S7a-b´´, respectively. Scale bar 50 μm

Individual FRMD6-positive fibers could be detected throughout the neuropil of this entire brain region and additionally in the circumventricular organs, i.e., the organon vasculosum laminae terminalis and the subfornical organ. Notable exceptions without detectable fibers were the part of the hippocampus and the basal ganglia complex, which were adjacent to the diencephalon. Occasionally, positive fibers were observed in the cingulate gyrus above the corpus callosum but not in the remaining cerebral cortex overlaying the diencephalic region. FRMD6-ir fibers were detectable in the entire hypothalamic area, i.e., periventricular and in the medial and lateral hypothalamus. Apart from labeled fibers running individually throughout the entire region, low-density to dense networks of FRMD6-positive terminals could be observed in the basal forebrain, the septal area, the hypothalamus and the dorsal diencephalon (Table 1). The densest fiber plexus was observed in the medial and lateral part of the median eminence, which was highlighted by intense FRMD6 immunofluorescence in all animals (Fig. 5a). Neuronal cell bodies with FRMD6 staining, however, were not seen, which was to be expected, as no pretreatment of the animals with intraventricular application of colchicine had been done. Therefore, the particular hypothalamic nucleus giving origin to the fibers to the median eminence could not be tracked down. Prominent tangles of FRMD6-positive fiber networks were additionally observed in brain areas, which are part of the dorsal diencephalic conduction system: the horizontal limb of the diagonal band of Broca, the intermediate part of the lateral septal nucleus, the stria medullaris and the lateral habenular nucleus (ESM, Fig. S7g-h´´). Fiber terminals were furthermore observed in midline thalamic nuclei, i.e., the paraventricular thalamic nucleus and what was regarded based on its topology as the centromedian thalamic nucleus.

**Table 1:**
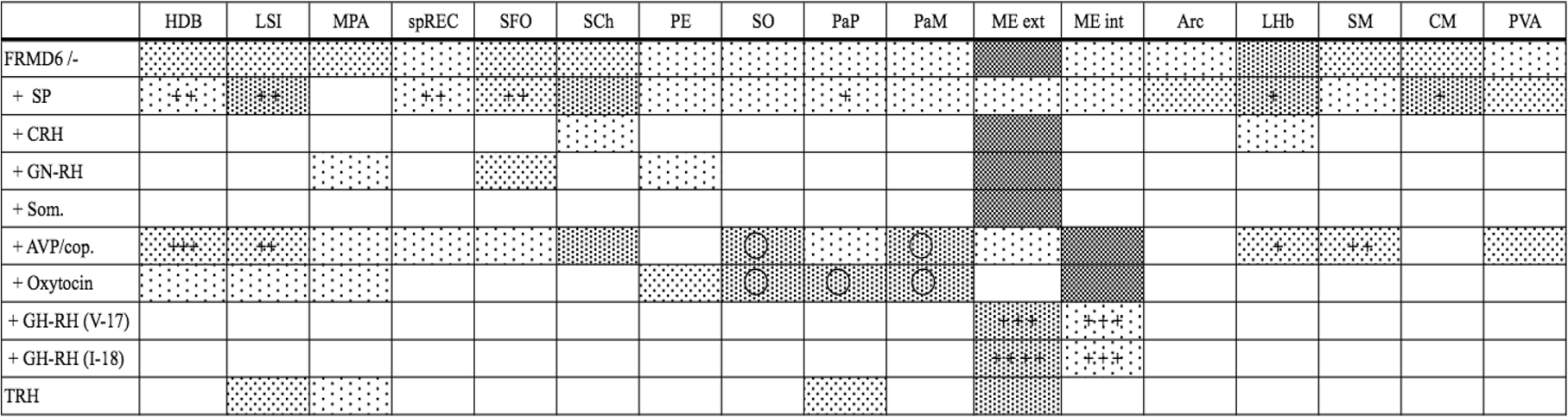
Colocalization of FRMD6 and hypothalamic neuropeptides in the rat brain. The table shows the results of the analyses of serial sections from the basal forebrain and diencephalon labeled with FRMD6 in combination with SP and the hypophysiotropic peptide hormones. The density of labeled nerve fibers in a specific region was staged semi-quantitatively into four categories and is displayed in increasing intensities of gray shades: (i) exclusively single fibers; (ii) low-density fiber network; (iii) network of fibers; and, ultimately, (iiii) dense network of fibers. The nomenclature used for different brain nuclei and regions is according to Paxinos and Watson (1998). The first row indicates the innervation density of FRMD6-ir fibers in specific diencephalic brain regions and the subsequent rows indicate the innervation densities of the respective neuropeptide in these same brain areas. Cells with black crosses indicate co-localization events in nerve fibers in double immunofluorescence experiments of FRMD6 with the respective neuropeptide. The quantity of fibers with detectable co-localization was semi-quantitatively staged into four categories and is indicated by increasing the numbers of crosses: (+) less than 25% of fibers show co-localization, (++) 25–50% co-localization, (+++) 50–75% co-localization, (++++) 75–100% co-localization. Cells marked by a circle represent regions, where neuronal cell bodies in addition to nerve fibers could be observed. *SP*, substance P; *CRH*, corticotropin-releasing hormone; *GN-RH*, gonadotropin-releasing hormone; *Som*, somatostatin; *AVP/Cop,* arginine vasopressin/copeptin; *GH-RH,* growth hormone-releasing hormone; *TRH*, thyroliberin; *HDB*, horizontal limb of the diagonal band of Broca; *LSi*, intermediate lateral septal nucleus; *MPA*, medial preoptic area; *sp REC*, supraoptic recess; *SFO*, subfornical organ; *SCh*, suprachiasmatic nucleus; *PE*, periventricular hypothalamic nucleus; *SO*, supraoptic nucleus; *PaP*, parvicellular part of the paraventricular hypothalamic nucleus; *PaM*, magnocellular part of the hypothalamic paraventricular nucleus; *ME ext.*, external zone of the median eminence; *ME int.*, internal zone of the median eminence; *Arc*, arcuate nucleus; *LHb*, lateral habenular nucleus; *SM*, stria medullaris; *CM*, central medial thalamic nucleus; *PVA*, paraventricular thalamic nucleus

Double and triple immunofluorescence were performed of FRMD6 in combination with antibodies against different neuropeptides raised in species other than rabbit to elucidate the co-occurring neuropeptides in FRMD6-ir fibers and terminals in the rat diencephalon and particularly the median eminence. As the median eminence receives only a sparse innervation by SP-containing nerve fibers, a co-localization of FRMD6 with a neuropeptide other than SP was suggested. The dense FRMD6 fiber plexus located in the external zone of the median eminence co-localized exclusively with growth hormone-releasing hormone (GH-RH) but not with any other hypophysiotropic hormone nor with SP. Single FRMD6/GH-RH double immunopositive fibers were also detected in intimate association with the specialized large capillary loops in the internal zone of the median eminence (Fig. 5a). We substantiated these findings by using two different antisera raised against human placental (hpGH-RH) and rat hypothalamic GH-RH (rhGH-RH). Human and rat GH-RH exhibit overall 30% non-homology but non-homologous amino acid substitutions between both peptides are clustered at the C-terminal end. As reported in the literature before, antisera raised against the N-terminus of hpGH-RH show cross-reactivity with rhGH-RH (Daikoku et al. 1985). We obtained identical results regardless which one of the two GH-RH antisera were used. We could not perform double staining of FRMD6 in combination with thyrotropin-releasing hormone (TRH), as no suitable antiserum from a species other than from rabbit was commercially available raised against the TRH tripeptide. However, by double immunofluorescence of TRH and GH-RH, it was confirmed that both hormones were released from entirely different nerve terminals in the median eminence and, therefore, colocalization of FRMD6 and TRH could be ruled out. Furthermore, reports in the literature confirm very little peptide co-localization among the hypophysiotropic hormones and none of TRH with GH-RH (Simmons and Swanson 2009). Therefore, the FRMD6-positive afferents to the external zone of the median eminence were co-localized exclusively with GH-RH.

In the forebrain, extensive colocalization of FRMD6 with arginine vasopressin (AVP) was detected in nerve fibers morphologically similar to terminal like fields in the diagonal band of Broca (Fig. 5b). Colocalization of AVP-positive fibers with FRMD6 was additionally observed in the lateral septum, the stria medullaris and to a lesser degree in the lateral habenular nucleus (Table 1). As in these brain areas, SP-containing fibers coexist; triple immunofluorescence with FRMD6, SP and AVP was deployed, to elucidate whether all three makers belonged to a single fiber population or whether SP and AVP occurred in disparate populations. Two separate fiber populations of about equal magnitude were found, one double immunopositive for FRMD6/AVP, the other double positive for FRMD6/SP. A third, minor population of FRMD6-ir fibers was neither positive for SP nor AVP (ESM, Fig. S7e-f´´´). In contrast, in the hypothalamus, co-localization of FRMD6 with AVP was neither observed in the supraoptic/paraventricular nuclei of the hypothalamo-neurohypophyseal system nor in the AVP-expressing fibers and neurons of the suprachiasmatic nucleus (ESM, Fig. S7c-d´´). Comparably, the paraventricular nucleus of the thalamus containing both FRMD6- and AVP-positive fibers did not show significant co-localization of both markers. Colocalization of FRMD6 with AVP was, therefore, restricted to a specific neural subsystem in the forebrain.

FRMD6-ir fibers were additionally observed in the pituitary gland with a discrepancy between the adeno- and neurohypophysis. In the adenophyophysis, FRMD6-ir fibers were double-stained for SP, while in the neurohypophysis, FRMD6-ir fibers were negative for SP (ESM, Fig. S7i-j´´).

### Endogenous FRMD6 colocalizes with preprotachykinin in vitro

**Fig. 6:**
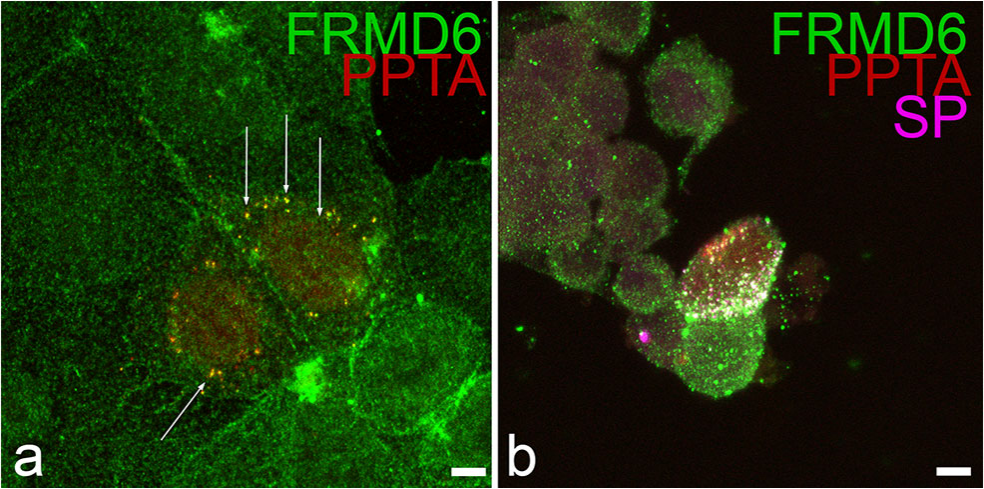
Co-localization of FRMD6 with PPTA. A PPTA red fluorescent fusion protein construct was expressed in MCF-7 (**a**) or AtT-20 (**b**) cell lines, respectively. **a** Double fluorescence of FRMD6-ir (green) and PPTA (red) detected by its red autofluorescence. In the center of the image, two rounded MCF-7 cells can be observed expressing PPTA. Yellow fluorescent granules (arrows) at the periphery of the two transfected cells indicate spots of co-localization of FRMD6 with PPTA. **b** Image of an AtT-20 cell culture with a PPTA-expressing cell in the center. Three channel overlay image of FRMD6-ir (green), SP-immunoreactivity (magenta) together with the PPTA fusion protein (red). Co-localization events result in white color. Extensive co-localization of FRMD6 with SP in granules near the right cell cortex is detectable in the PPTA expressing cell. The individual images of each fluorescence channel of Fig. 6 can be found in ESM, Fig. S8a-b´´´, respectively. Scale bar 5 μm

In contrast to classical neurotransmitters, which are packaged in small synaptic vesicles, neuropeptides are stored in large dense-core vesicles (LDCV). They arise by sorting the precursor molecules of the respective neuropeptide into the regulated secretory pathway. The inactive precursors are subsequently processed into the actual bioactive forms by endoluminal cleavage involving specific carboxypeptidases. Recently, it has been shown that the expression of the SP precursor molecule preprotachykinin A (PPT-A) even in non-neuronal cells induces the synthesis of LDCVs, which recruit lipids and other proteins, e.g., the δ-opiod receptor, to their surface mimicking neuronal SP positive vesicles (Guan et al. 2006). As we found in tissues of three different species a close association of endogenous FRMD6-ir with SP at neuronal vesicles of varicose nerve fibers, we explored whether this correlation was reproducible in cultured non-neuronal cell lines with induced LDCVs. Therefore, we expressed a PPT-A red fluorescent protein fusion construct in cultured AtT20 and MCF-7 cells. The AtT20 cell line is derived from a mouse pituitary tumor and retains many characteristics of hormone secreting cells. This cell line has, therefore, often been used a model system for the study of peptide hormone secreting cells. By SP immunofluorescence, it could be confirmed that transfection of the PPT-A expression construct led to SP-positive cytoplasmic vesicles in transfected MCF-7 and AtT20 cells. Double immunofluorescence with the FRMD6 antibody revealed that the endogenous FRMD6 protein in MCF-7 and particularly in AtT20 cell cultures was recruited to the induced LDCVs and colocalized closely with SP similar to the observations in neural tissues (Fig. 6).

## Discussion

The antibody, used in this study, was raised against an epitope comprising 24 aa in the N-terminally located FERM domain of rat FRMD6. Although FERM domains have a high degree of sequence homology, which is an obstacle to the development of specific antibodies, alignment of the epitope region with homologous FERM domains shows a significant sequence variation of FRMD6 in the epitope region with an additional insertion of two hydrophilic amino acids (ESM, Fig. S3c). Immunofluorescence analysis of cell lines using this antibody showed a granular or vesicular staining at the cell periphery. In contrast, prototypical FERM domain–containing proteins, e.g., ezrin or merlin, are associated with the plasma membrane and particularly with plasma membrane protrusions like microvilli, lamellipodia and filopodia (Bretscher et al. 2002). Characteristic staining of plasma membrane protrusions, which could be interpreted as a sign of cross-reactivity with other FERM proteins, however, was not observed. Furthermore, the FRMD6 antiserum proved capable of immunoprecipitating the FRMD6 full-length protein in extracts of cell cultures. In neuronal tissues, we found FRMD6 labeling exclusively associated with nerve fibers containing different neuropeptides, e.g., in the spinal cord FRMD6 staining, we could clearly differentiate between afferent nerve fibers containing the neuropeptides SP and CGRP, which coexist in secretory vesicles (Salio et al. 2006) and those binding to IB4, two non-overlapping unmyelinated fiber populations to the same target area in the dorsal horn of the spinal cord (Guo et al. 1999) (ESM, Fig. S4). In this context, it is very interesting that the FERM protein ezrin is similarly found in only a subset of neuronal sensory projections, i.e., to specific laminae of the optic tectum (Takahashi et al. 1999). However, by close examination, ezrin has also been found in glial cells, i.e., in astrocyte processes of the rat hippocampus (Derouiche and Frotscher 2001). Whether FRMD6 staining additionally occurs in glial elements as well was not the focus of this study and has to be clarified in future studies.

Surprisingly, FRMD6-ir was not universally associated with LDCVs nor was restricted to LDVs containing a particular neuropeptide or belonging to a single neural subsystem. The quantitative most significant association was the association of FRMD6 with SP in varicose nerve fibers in the peripheral and to a smaller part in the central nervous system, e.g., in the basal forebrain or septum. This proved not to be an invariable correlation; however, as in a number of instances, e.g., in the median eminence, neurohypophysis or arcuate nucleus, SP-positive fibers without FRMD6-ir occurred. In the hypothalamic-hypophysiotropic system FRMD6-ir was exclusively associated with GH-RH releasing terminals, while nerve fibers containing any of the remaining hypophysiotropic peptide hormones were not co-labeled with FRMD6. As a corollary, the FRMD6 protein is not an essential requirement in the biogenesis or secretion of neuropeptidergic vesicles, like, e.g., SNARE or Rab proteins but a potential co-factor of LDCVs in specific neurons. Recently, the classic view of LDCVs as molecularly homogenous in the composition of their adaptor proteins and in their trafficking mode has been challenged and a cell-type dependency established (Ramamoorthy et al. 2011; Ramirez-Franco et al. 2016).

The function of the FRMD6 wildtype protein in mature postmitotic neurons is unknown. The FRMD6 protein possesses no signal peptide for entering the regulated secretory pathway nor is processed from prohormone-like classical neuropeptides. Association of FERM domain–containing proteins with vesicles of the endomembrane system, like clathrin-coated vesicles, phagosomes, endosomes, or melanosomes, always occurs from the cytoplasmic side, where they mediate the maturation and trafficking of the respective vesicles by organizing the underlying actomyosin cytoskeleton (Erwig et al. 2006; Schwander et al. 2009; Semenova et al. 2009; Chirivino et al. 2010; Muriel et al. 2015).

FRMD6 has been identified as the closest vertebrate orthologue to the protein expanded in Drosophila, which is an upstream regulator of the growth-promoting Hippo signal transduction pathway. The function of FRMD6 has, therefore, been mainly investigated in the context of cell proliferation or tumor suppression (Angus et al. 2012). But independent from its effect on cell proliferation, FRMD6 and expanded regulate apical plasma membrane domain size. In Drosophila epithelial cells, expanded acts as a sensor of apical domain stretch and adjusts the cellular response to the mechanical strain by the Hippo pathway (Fletcher et al. 2018). Apical domain size and membrane tension play a prominent role not only in morphogenesis and regulation of cell proliferation by the Hippo pathway but also additionally in synaptic vesicle exocytosis and exocytosis-endocytosis coupling (Lou 2018). FRMD6 has been shown to regulate the constriction of circumferential actomyosin cables at adherens junctions and thereby the size of the apical domain (Ishiuchi and Takeichi 2011). The cortical actomyosin network, however, additionally has a function in the trafficking of secretory vesicles to their exocytotic release sites, adjustment of membrane tension and in the dynamics of the fusion pore (Meunier and Gutiérrez 2016). A functional role for FRMD6 in the latter, however, has yet to be established.

In three independent neuroimaging genetics association studies, a highly significant correlation of the FRMD6 gene to Alzheimer’s disease and mild cognitive impairment was discovered and this correlation was additionally validated by a genome-wide and gene-based association study in a large patient cohort (Hong et al. 2012; Shen et al. 2014). In this respect, it is noteworthy that we did not find any FRMD6-ir fibers in the hippocampus, the brain area predominantly implicated in Alzheimer’s disease pathogenesis. Staining of brain areas linked to memory formation, however, was observed in case of the FRMD6-ir fibers in Broca’s diagonal band and in the septum. These areas receive a dense reciprocal connection with the hippocampus and have among others been attributed a pathogenetic role in Alzheimer’s disease (Buzsáki 2002; Landgraf et al. 2003; Stoop 2012; Borbély et al. 2013; Van Dam et al. 2013).

## Electronic supplementary material

ESM 1 (PDF 14115 kb)

ESM 2 (PDF 8103 kb)

ESM 3 (PDF 7385 kb)

ESM 4 (PDF 7270 kb)

ESM 5 (PDF 15536 kb)

ESM 6 (PDF 9491 kb)

ESM 7 (PDF 25.2 mb)

ESM 8 (PDF 45453 kb)

ESM 9 (PDF 38895 kb)

ESM 10 (PDF 18738 kb)

ESM 11 (DOCX 33 kb)
