# Supplementary figures and images for: FERM domain–containing protein 6 identifies a subpopulation of varicose nerve fibers in different vertebrate species
Source: Cell Tissue Res. 2020 Mar 21;381(1):13–24. doi: 10.1007/s00441-020-03189-7 (PMC7306050; doi:10.1007/s00441-020-03189-7)

Figure S1

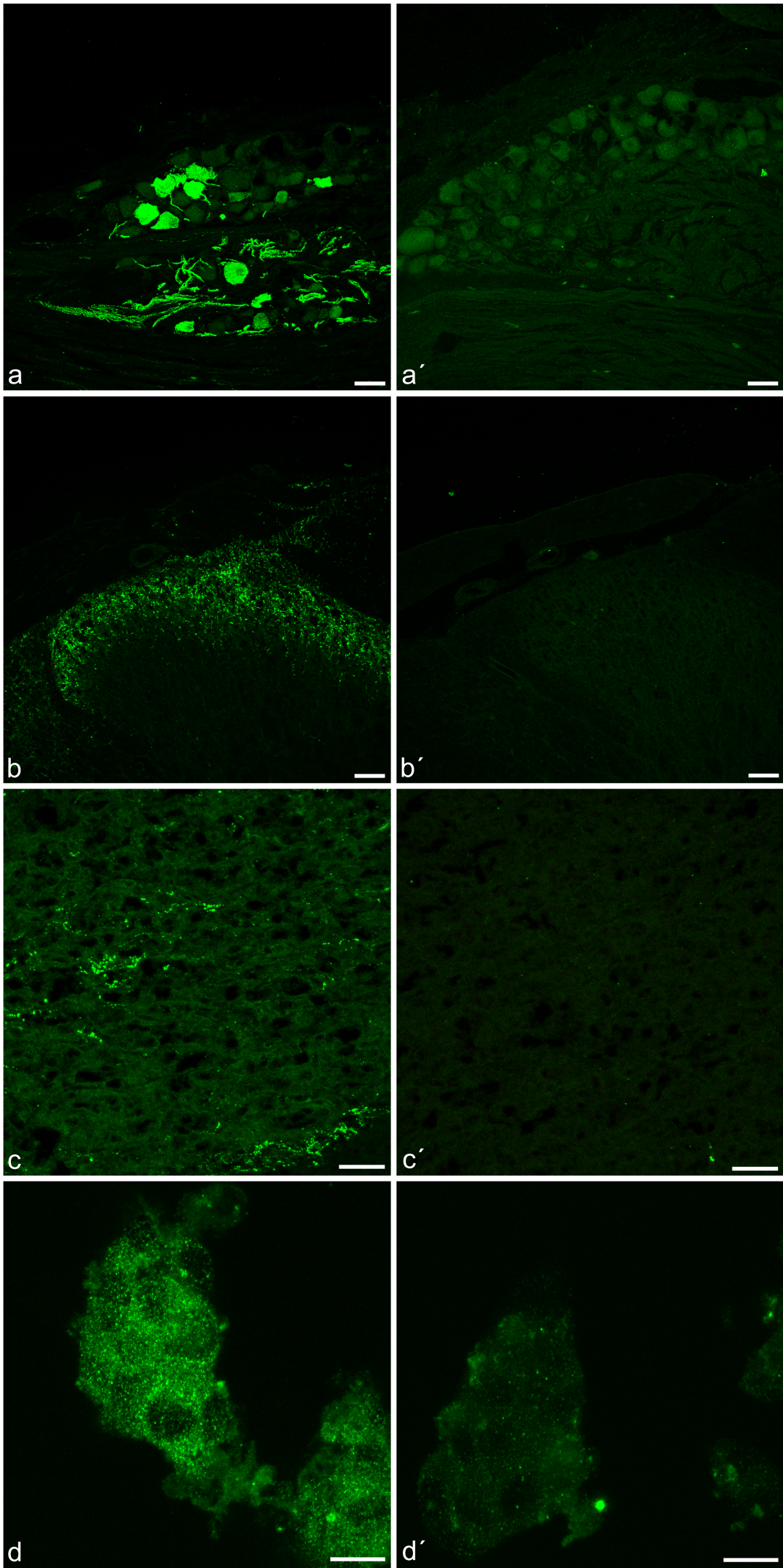

Supplement: Supplementary file 1 — (PDF 14115 kb) [file 441_2020_3189_MOESM1_ESM.pdf]

Figure S2

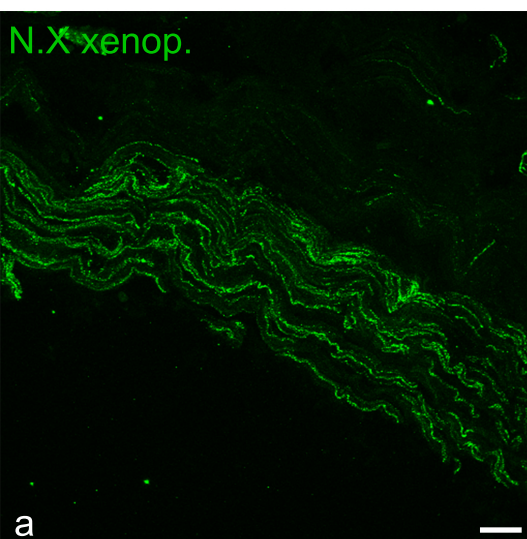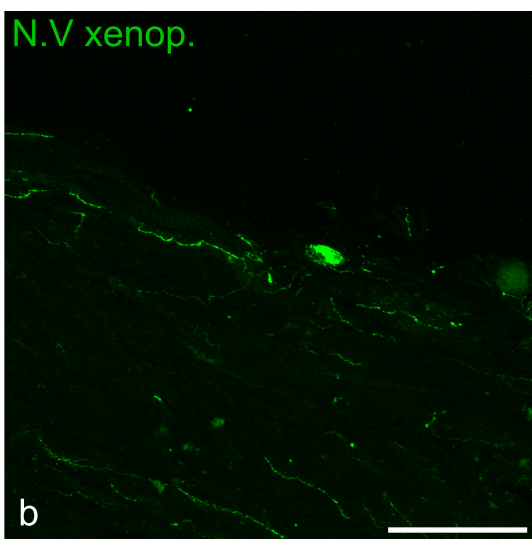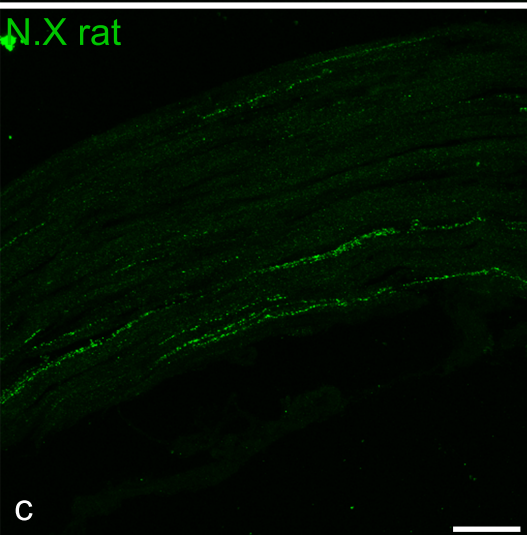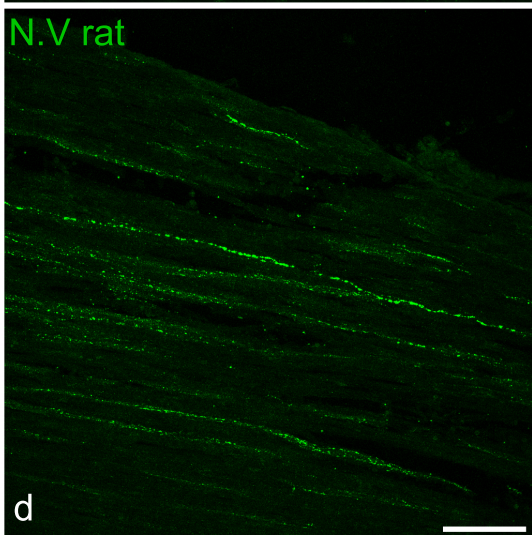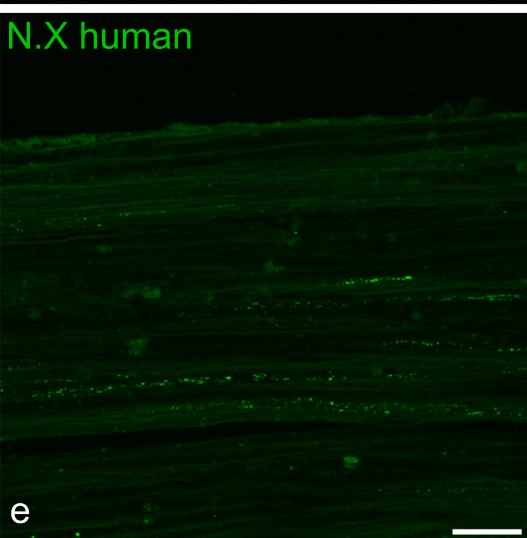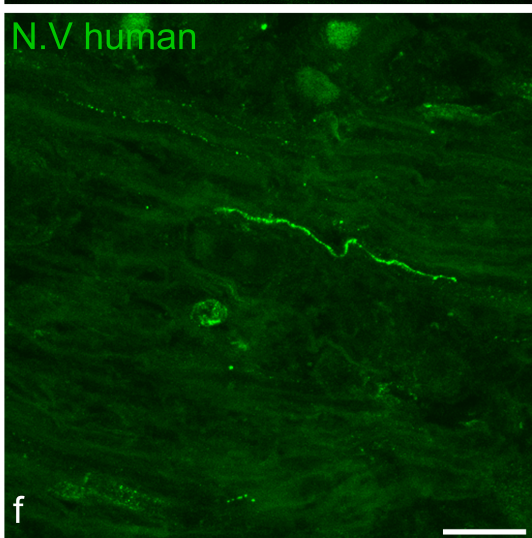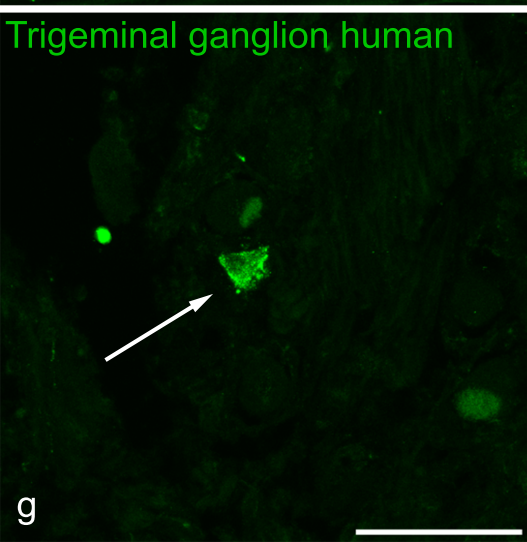

Supplement: Supplementary file 2 — (PDF 8103 kb) [file 441_2020_3189_MOESM2_ESM.pdf]

Figure S4

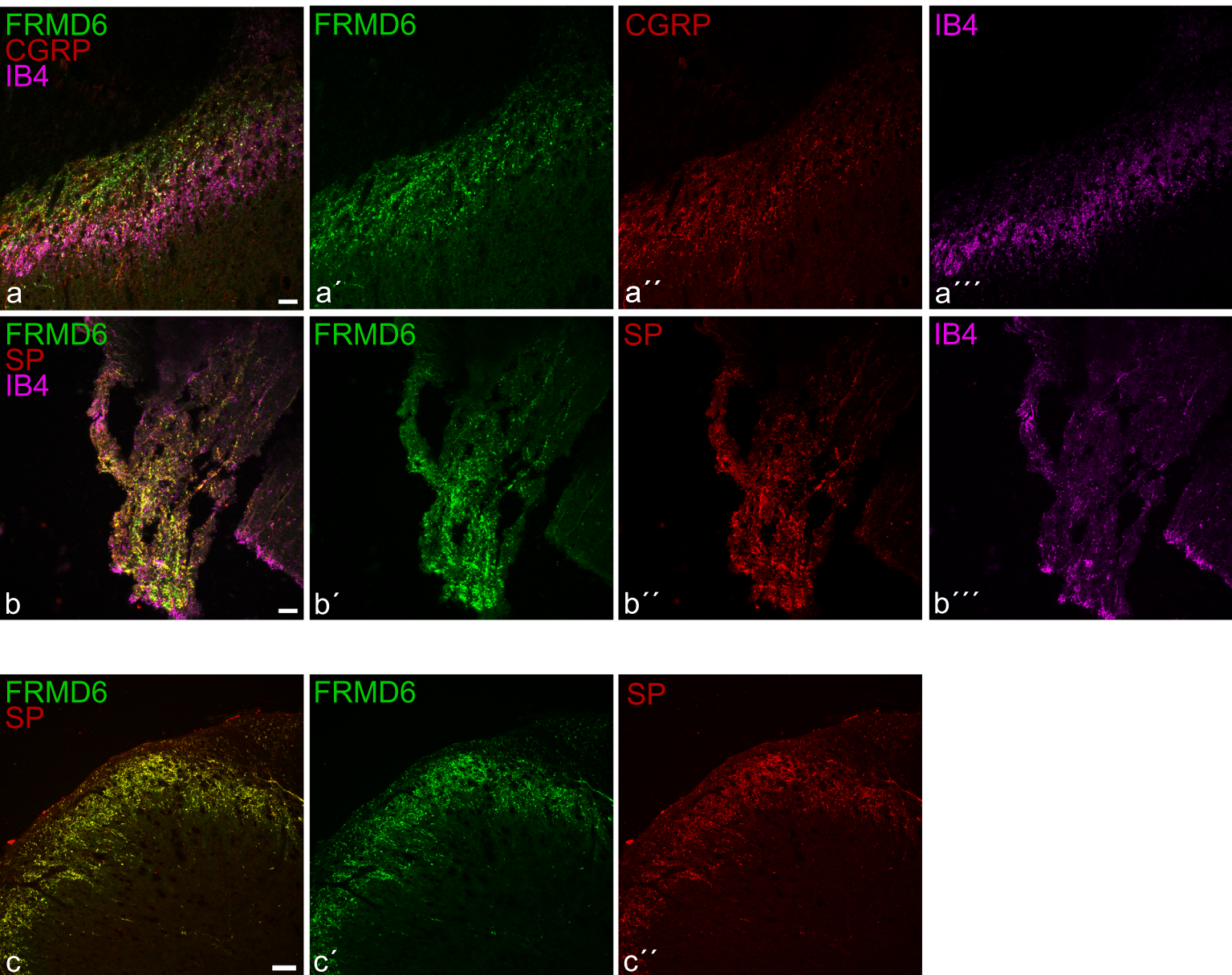

Supplement: Supplementary file 4 — (PDF 7270 kb) [file 441_2020_3189_MOESM4_ESM.pdf]

Figure S5

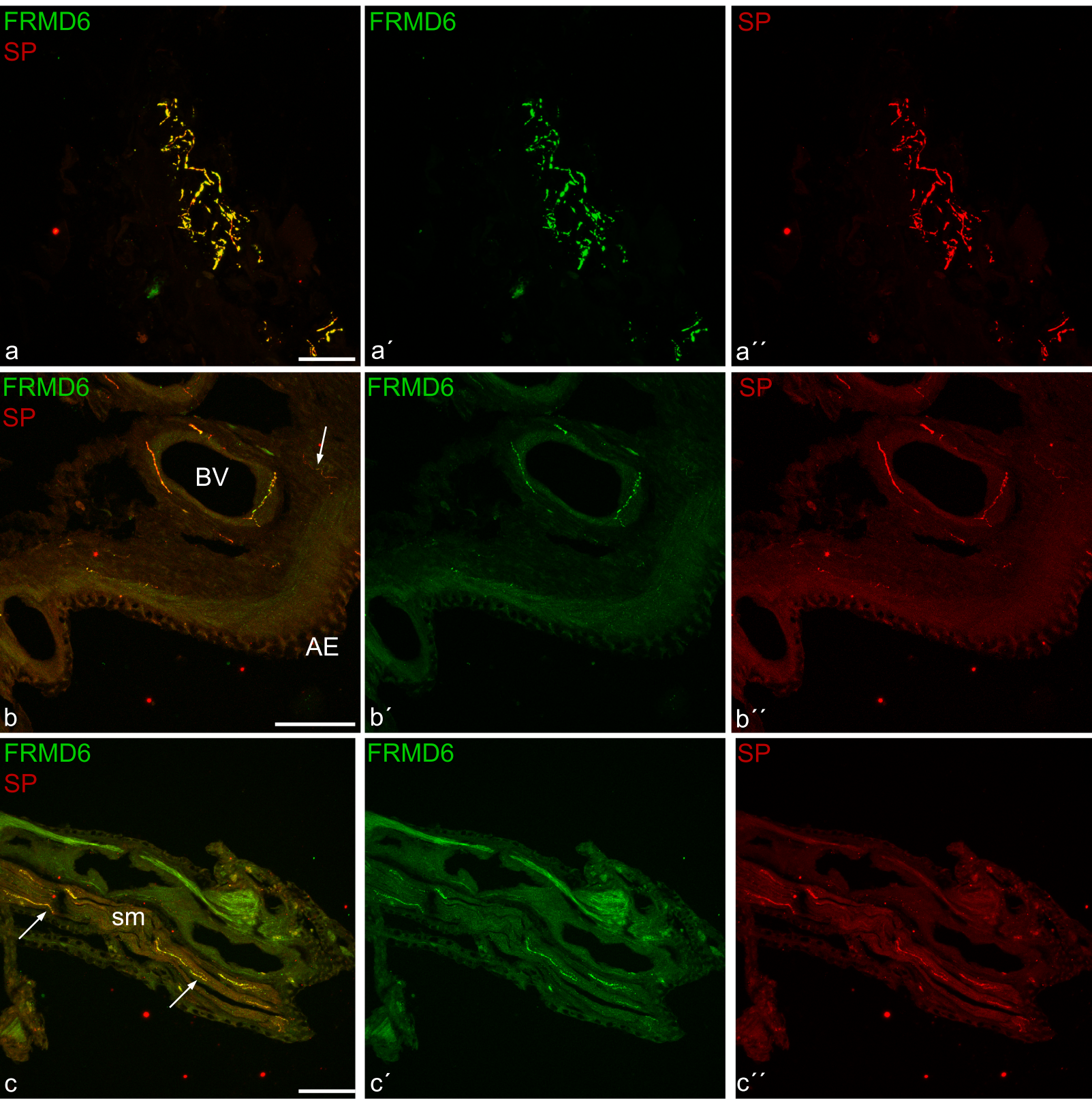

Supplement: Supplementary file 5 — (PDF 15536 kb) [file 441_2020_3189_MOESM5_ESM.pdf]

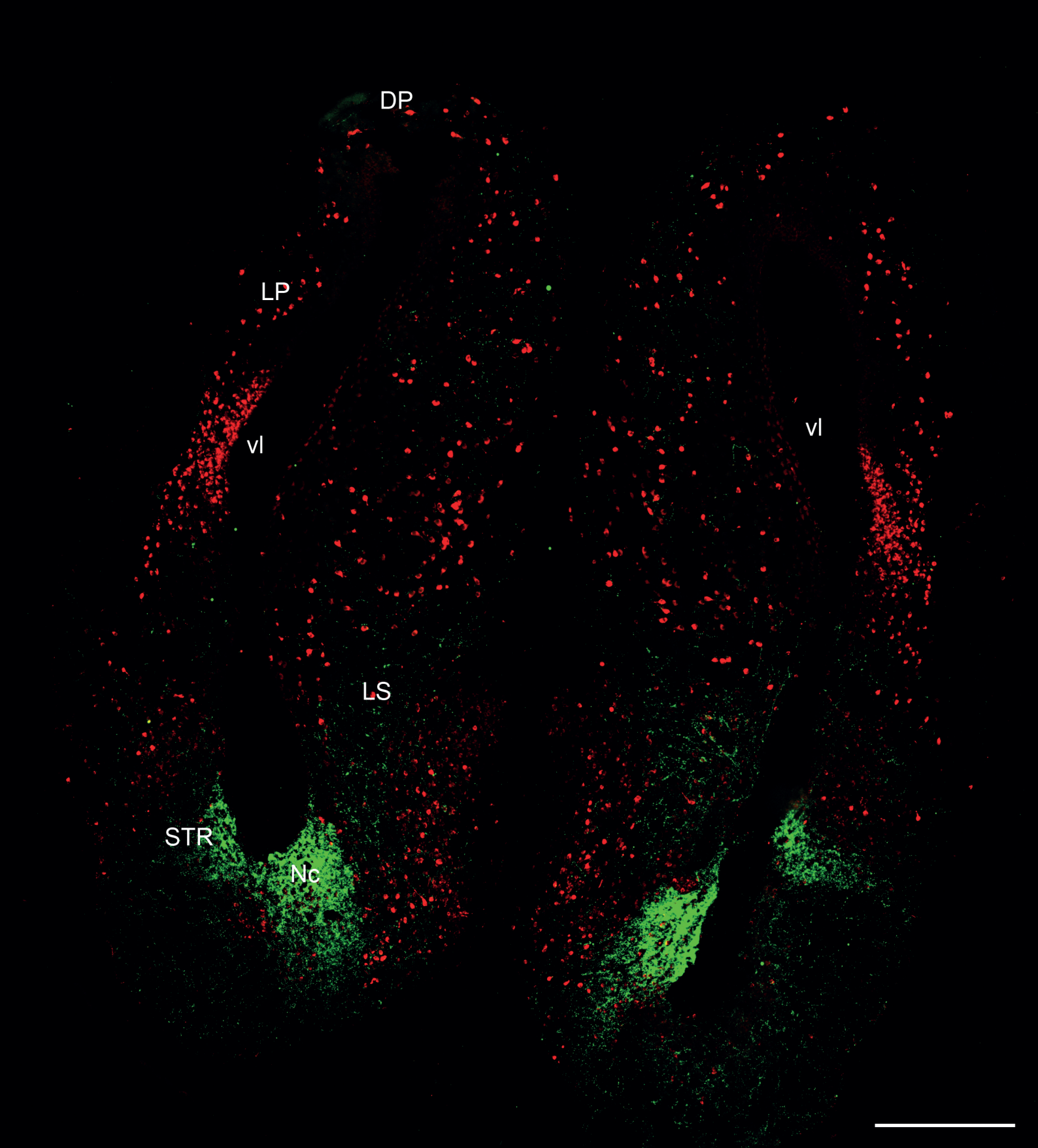

Supplement: Supplementary file 6 — (PDF 9491 kb) [file 441_2020_3189_MOESM6_ESM.pdf]

Figure S7 a-d

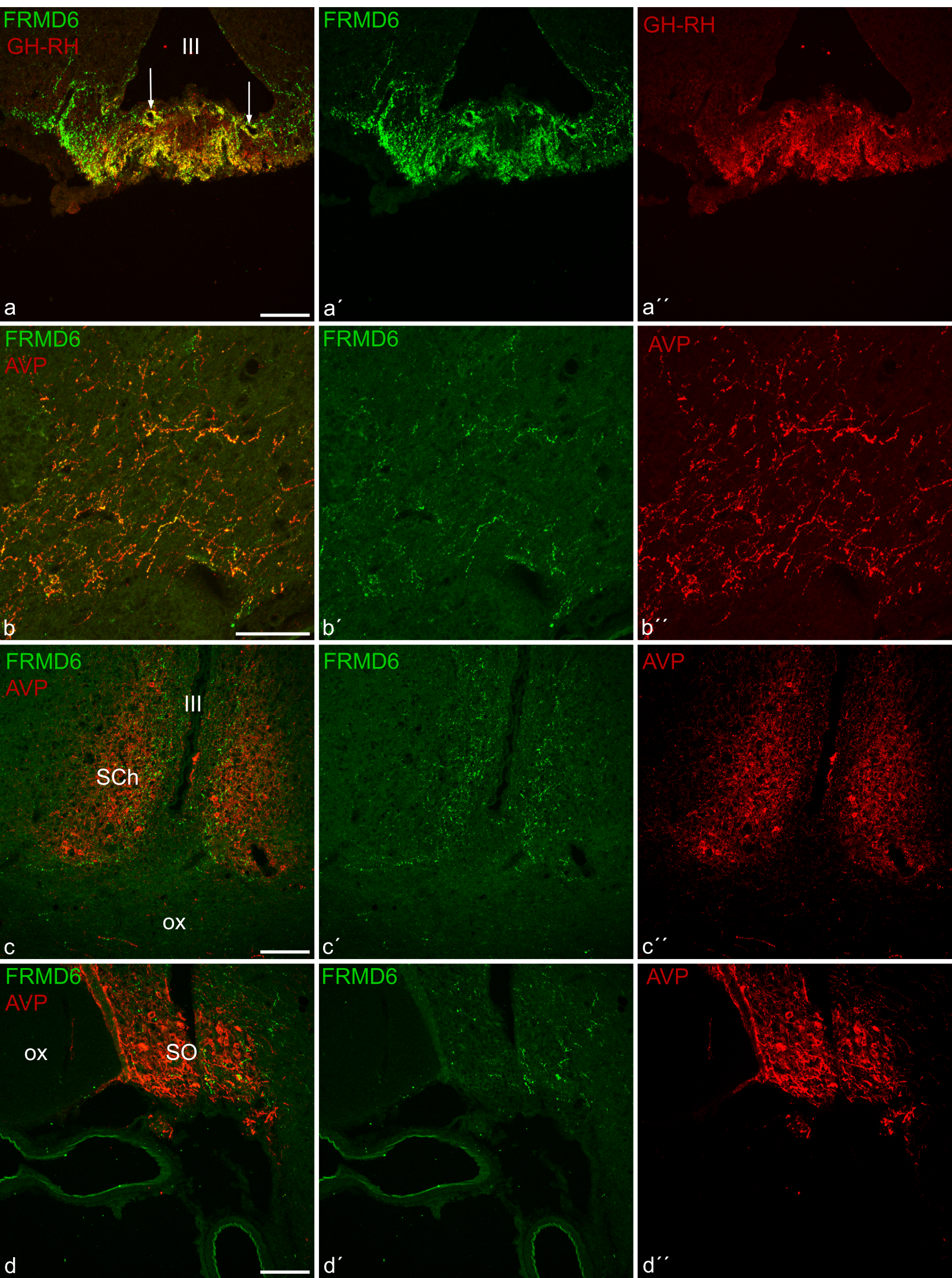

Supplement: Supplementary file 7 — (PDF 25.2 mb) [file 441_2020_3189_MOESM7_ESM.pdf]

Figure S7 e-f

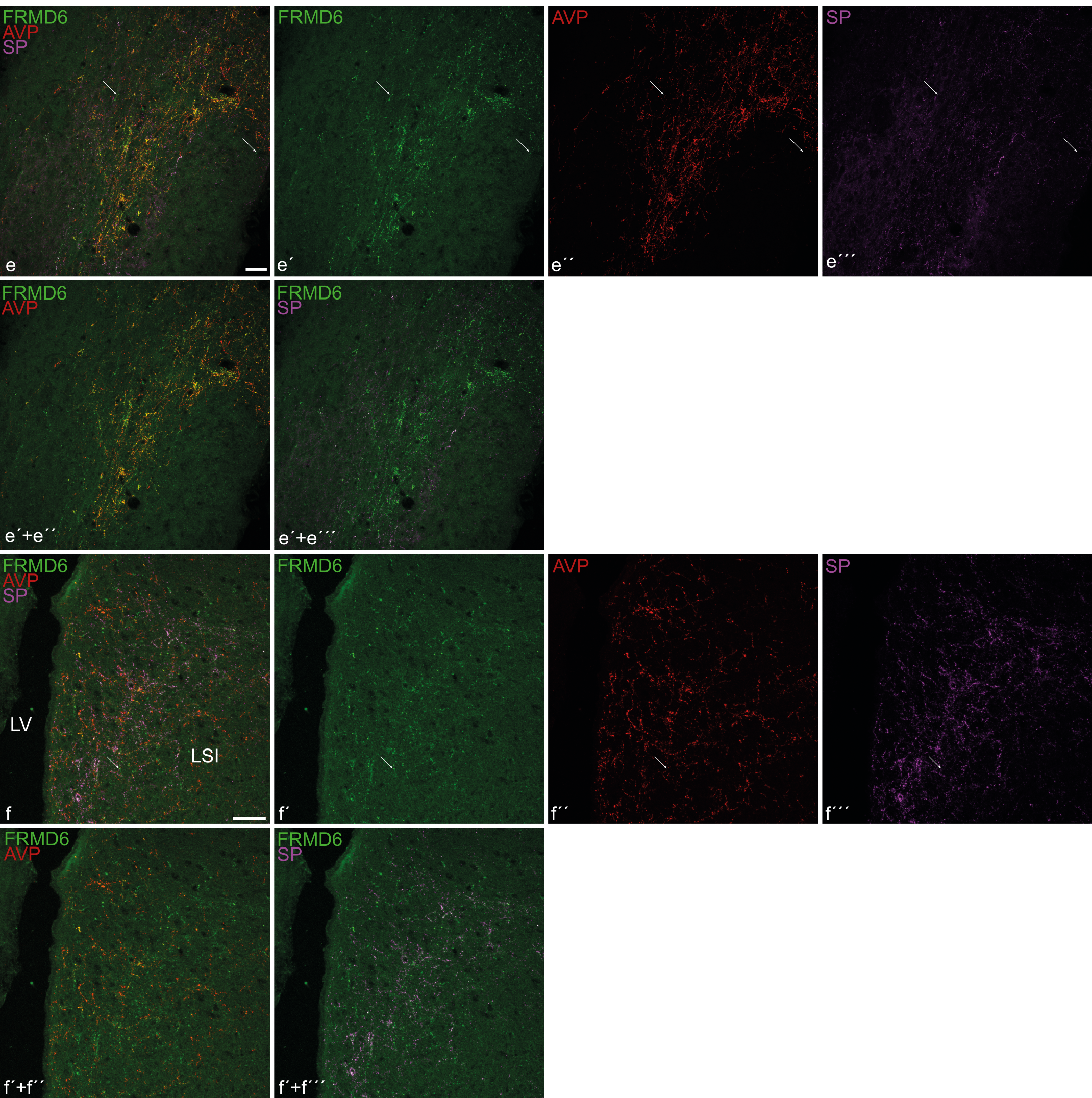

Supplement: Supplementary file 8 — (PDF 45453 kb) [file 441_2020_3189_MOESM8_ESM.pdf]

Figure S7 g-j

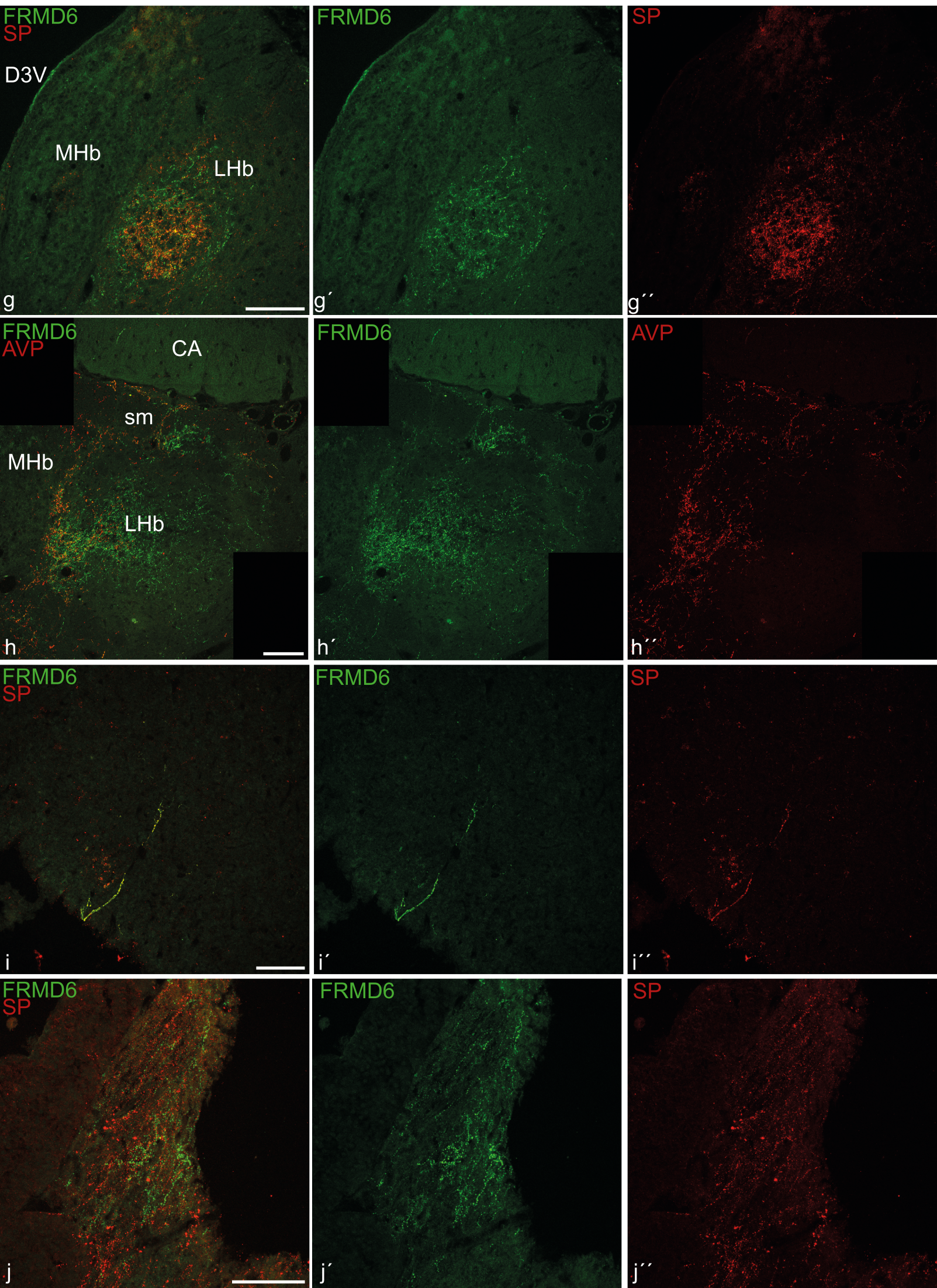

Supplement: Supplementary file 9 — (PDF 38895 kb) [file 441_2020_3189_MOESM9_ESM.pdf]

Figure S8

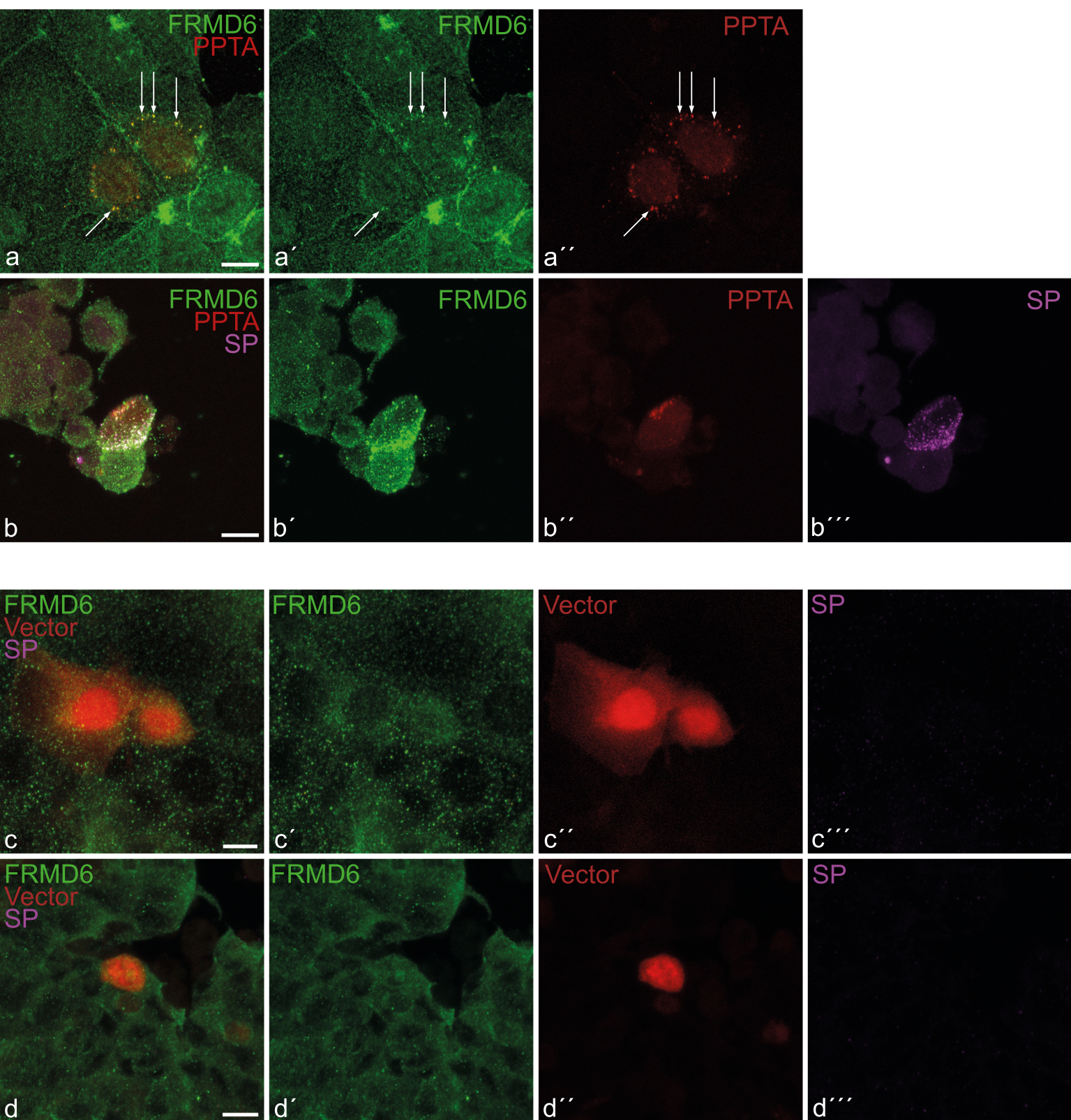

Supplement: Supplementary file 10 — (PDF 18738 kb) [file 441_2020_3189_MOESM10_ESM.pdf]
