# Supplementary material for: FERM domain–containing protein 6 identifies a subpopulation of varicose nerve fibers in different vertebrate species
Source: Cell Tissue Res. 2020 Mar 21;381(1):13–24. doi: 10.1007/s00441-020-03189-7 (PMC7306050; doi:10.1007/s00441-020-03189-7)
Supplement: Supplementary file 3 — (PDF 7385 kb) [file 441_2020_3189_MOESM3_ESM.pdf]

Figure S3

a

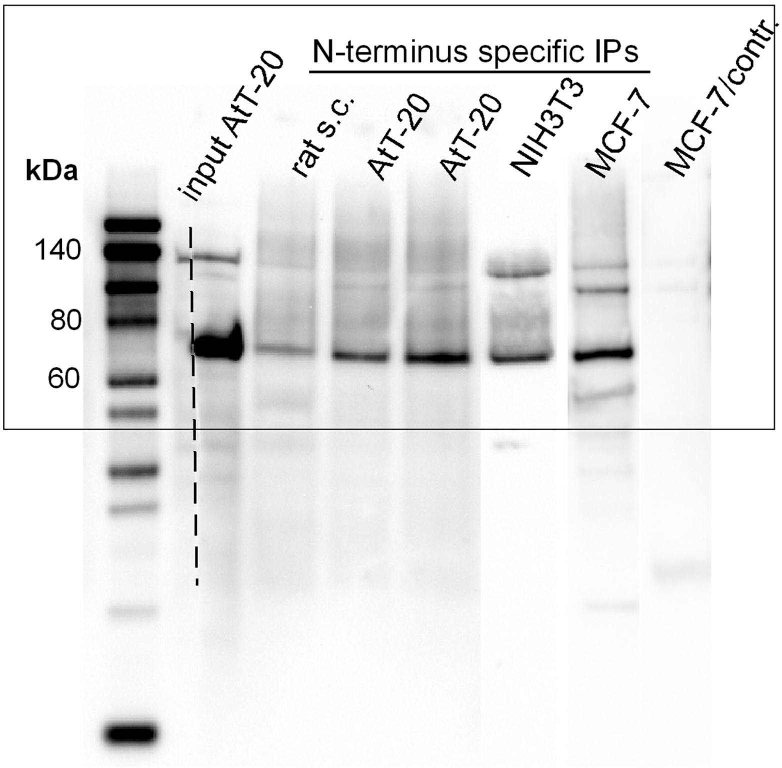
N-terminus specific IPs
kDa
140
80
60
input AtT-20
rat s.c.
AtT-20
AtT-20
NIH3T3
MCF-7
MCF-7/contr.

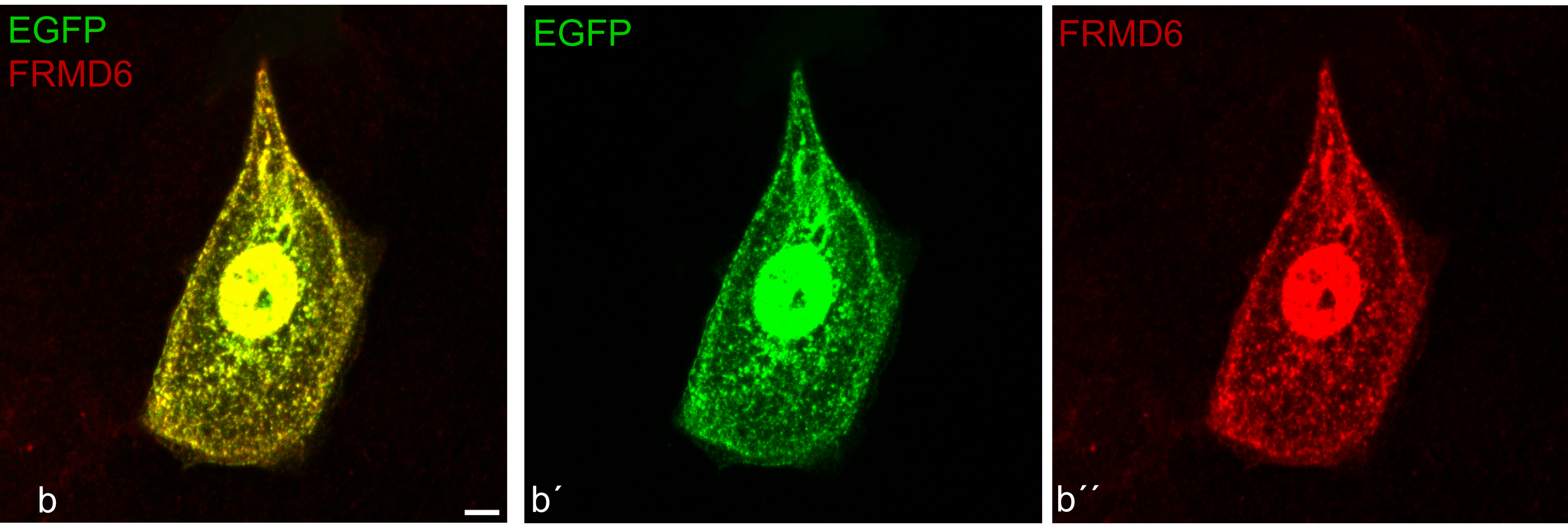
EGFP
FRMD6
b
EGFP
b´
FRMD6
b´´

c

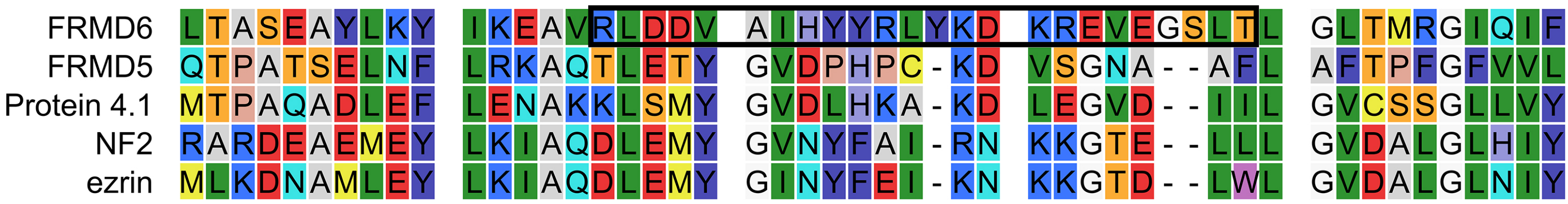
FRMD6 LTASEAYLKY IKEAVRLDDV AIHYYRLYKD KREVEGSLTL GLTMRGIQIF
FRMD5 QTPATSELNF LRKAQTLETY GVDPHPC-KD VSGNA--AFL AFTPFGFVVL
Protein 4.1 MTPAQADLEF LENAKKLSMY GVDLHKA-KD LEGVD--IIL GVCSSGLLVY
NF2 RARDEAEMEY LKIAQDLEMY GVNYFAI-RN KKGTE--LLL GVDALGLHIY
ezrin MLKDNAMLEY LKIAQDLEMY GINYFEI-KN KKGTD--LWL GVDALGLNIY
