# Supplementary material for: FERM domain–containing protein 6 identifies a subpopulation of varicose nerve fibers in different vertebrate species
Source: Cell Tissue Res. 2020 Mar 21;381(1):13–24. doi: 10.1007/s00441-020-03189-7 (PMC7306050; doi:10.1007/s00441-020-03189-7)
Supplement: Supplementary file 11 — (DOCX 33 kb) [file 441_2020_3189_MOESM11_ESM.docx]

Cell & Tissue Research

FERM domain containing protein 6 identifies a subpopulation of varicose nerve fibers in different vertebrate species

Josefa Beck and Michael Kressel

**Supplementary Table S1**

List of all primary antibodies used in the study. Neurophysin I represents the prohormone of oxytocin and can be used as a maker protein for oxytocin containing neurons. Copeptin is the C-terminal cleavage product of the prohormone for AVP (provasopressin, neurophysin II). An antiserum raised against the copeptin moiety of neurophysin II is, therefore, a marker for AVP releasing nerve fibers (Cubois-Dauphin and Zakarian 1987).

| Name | Antigen | Species | Host | Dilution | Source |
| --- | --- | --- | --- | --- | --- |
| FRMD6 (K-13) | internal region N-terminal half of the protein, aa 217-240 | rat | rabbit | 1:1000 | Santa Cruz Biotechnology (SCBT):  sc-138006 |
| FRMD6 | C-terminus aa 446-580 | human | rabbit | 1:4000 | Sigma Aldrich |
| Substance P | aa 1-11 | rat | g.p. | 1:200 | Novus Biologicals |
| Substance P (N-18) | full-length | human | goat | 1:200 | SCBT: sc-9758 |
| GnRH I | GnRH1 rat | rat | mouse | 1:250 | SCBT: sc-32292 |
| Oxytocin (M-15) | Neurophysin I C-terminus | mouse | goat | 1:1000 | SCBT: sc-7810 |
| Vasopressin | Copeptin C-terminus | mouse | goat | 1:500 | SCBT: sc-7812 |
| CRF | CRF C-terminus | human | goat |  | SCBT:sc-1759 |
| GH-RH (V17) | GH-RH N-terminus | human | goat | 1:500 | SCBT: sc-10280 |
| GH-RH (I-18) | GH-RH C-terminus | rat | goat | 1:200 | SCBT: sc-10283 |
| Somatostatin | somatostatin | human | rat | 1:200 | SCBT: sc-47706 |
| Thyrotropin releasing hormone | Thyrotropin releasing hormone |  | rabbit | 1:200 | Acris |
| HuC | HuC, aa 265-287 | human | mouse | 1:1000 | SCBT: sc-515624 |
| Griffonia Simplicifolia Isolectin B4 (I-B4) |  |  | Lectin | 1:200 | Vector Laboratories |

**Supplemantary Tables S2-4** Results of immunofluorescence staining of rat (Table S2), xenopus (Table S3) and human (Table S4) cranial nerves. Cranial nerves (II-XII) in a representative number of animals and humans (n) were cut in toto into cryosections and stained for FRMD6. The nerve fiber density was staged semiquantitatively into four categories: Ø: no stained fibers detectable in the respective nerve; (X): a single questionable fiber per nerve in at least one animal; X: 1-3 positive fibers/nerve; XX: in average ≥ two FRMD6-ir nerve fibers per immunohistochemical section; XXX: an average of ≥ 5 FRMD6 positive nerve fibers/immunohistochemical section. As the overall FRMD6 staining intensity varied interindividually between rats, the last column in Table S2 shows the percentage of animals, in which the semiquantitative classification of the second column is applicable. The residual animals were classified into a lower category. nd (not determined).

**Table S2**

| Rat | degree of positive fibers | n | percentage |
| --- | --- | --- | --- |
| optic n. | $\emptyset$ | 2 | 0% |
| oculomotor n. | $\emptyset$ | 2 | 0% |
| trochlear n. | $\emptyset$ | 2 | 0% |
| trigeminal n. | XXX | 4 | 75% |
| abducens n. | $\emptyset$ | 2 | 0% |
| facial n. | $\emptyset$ | 2 | 0% |
| vestibulocochlear n. | $\emptyset$ | 2 | 0% |
| glossopharyngeal n. | XXX | 4 | 100% |
| vagus n. | XXX | 9 | 100% |
| accessory n. | (X) | 5 | 20% |
| hypoglossal n. | $\emptyset$ | 3 | 0% |

**Table S3**

| Xenopus | degree of positive fibers | n |
| --- | --- | --- |
| optic n. | $\emptyset$ | 1 |
| oculomotor n. | $\emptyset$ | 1 |
| trochlear n. | nd | nd |
| trigeminal n. | xx | 2 |
| abducens n. | nd | nd |
| facial n. | $\emptyset$ | 1 |
| vestibulocochlear n. | $\emptyset$ | 1 |
| glossopharyngeal n. | $\emptyset$ | 1 |
| vagus n. | xxx | 3 |

**Table S4**

| human | degree of positive fibers | n |
| --- | --- | --- |
| optic nerve | nd | nd |
| oculomotor n. | nd | nd |
| trochlear n. | nd | nd |
| trigeminal n. | x | 2 |
| abducens n. | nd | nd |
| facial n. | nd | nd |
| vestibulocochlear n. | nd | nd |
| glossopharyngeal n. | $\emptyset$ | 1 |
| vagus n. | x | 2 |
| accessory n. | $\emptyset$ | 2 |
| hypoglossal n. | nd | nd |

**Table S5** Ganglia of cranial nerves IX-XI (jugular, nodose and petrosal ganglion) were removed en bloc from the jugular foramen of the cranial base and sectioned into a total of 44 cryosections with a thickness of 16 µm and double stained for both FRMD6 and SP. All labelled neurons in all sections were counted in both fluorescence channels and categorized as either singly positive for either SP or FRMD6 or double positive for both FRMD6 and SP. Neurons were analysed exclusively in the plane of the cell nucleus, to rule out repeated counting of neurons expanding over adjacent cryosections. For the delimitation of the jugular vs. the nodose vagal ganglion the criteria of Helke and Hill (Neuroscience 26: 539-551) were used, but only a few positive neurons were found in the nodose ganglion, almost all at its cranial border next to the jugular ganglion. The table shows the number of positive neurons in each category. The average number of SP positive neurons/section was 7.3 for the jugular ganglion, 0.6 for the nodose and 1.6 for the petrosal ganglion. All FRMD6-ir neurons were double positive for SP, which indicates a unique subpopulation of FRMD6/SP positive neurons in the jugular ganglion.

| Immunoreactivity | Jugular  ganglion | Nodose  Ganglion | Petrosal  Ganglion |
| --- | --- | --- | --- |
| SP + FRMD6 | 292 | 22 | 45 |
| SP | 303 | 11 | 31 |
| FRMD6 | 0 | 0 | 0 |

**Supplementary Figures S1-S8**

**Figure S1** Preabsorption control experiments in different species and tissues. Positive (a, b, c, d) and preabsorption control slides (a´, b´, c´, d´) were processed in parallel except that for the preabsorption control slides the primary antibody was incubated with the homologous peptide antigen in a 1: 10 ratio by weight prior to its application. Images of positive and preabsorption controls were recorded successively by confocal microscopy with the same settings for contrast and brightness without any secondary image processing. Across all species and tissues the FRMD6 antiserum specifically labels varicose nerve fibers (a, c), nerve terminals (b) and neuronal cell bodies (a), whereas only background staining is detectable in the preabsorption controls. a, a´ xenopus NIX and NX ganglion complex. b, b´ rat dorsal horn. c, c´ infundibulum of rat neurohypophysis. d, d´ in At-T20 cell lines a punctate and granular staining can be seen. After preabsorption the granular staining can no longer be observed, but the punctate cytoplasmic staining persisted to some extent (d´). Scale bar 10 µm

**Figure S2** FRM6-ir nerve fibers in the cranial nerves of three different species. A segment of the vagal nerve in xenopus (a), rat (c) and human (e) is shown, as well as of the trigeminal nerve in xenopus (b), rat (d) and human (f). In all three species FRMD6 labels a comparable subpopulation of varicose nerve fibers. (g) Human trigeminal ganglion stained with the FRMD6 antibody. The arrow points to a FRMD6-ir ganglion cell. Scale bar 100 µm

**Figure S3** (a) Full scan of the Western blot presented in Fig. 3. Box indicates the area displayed in Fig. 3. (b-b´´) MCF-7 cell transfected with a fusion protein construct comprising EGFP and FRMD6 aa 217-240 fused to the C-terminus of EGFP. (b) Overlay image of the green and red channel demonstrates complete co-localization of both channels resulting in yellow colour. (b´) Green channel displaying EGFP detected by its autofluorescence. (b´´) The red channel shows FRMD6-ir detected by the N-terminus directed antiserum and a secondary Alexa555 antibody. Scale bar 5 µm (c) Amino acid sequence alignment of a section of the FRMD6 FERM domain with homologous proteins. The section comprises the epitope detected by the FRMD6 antiserum (bracketed) and the region immediately surrounding it. Shown sequences are derived from rat FRMD6 aa 202-240 (F1LR29), FRMD5 aa 186-232 (Q7Z6J6), protein4.1 aa 377-423 (P11171), neurofibromatosis type 2 (NF2) aa 198-244 (P35240) and ezrin aa 182-203 (P15311). The numbers in parentheses are the UniProt knowledgebase identifiers of the respective protein sequences.

**Figure S4** Overlay images (a,b) and each individual fluorescence channel (a´-b´´´) constituting the respective overlay image. (a-a´´´) Triple immunofluorescence staining of FRMD6 (a´), CGRP (a´´) and I-B4 (a´´´) of the rat spinal cord dorsal horn. FRMD6 and CGRP co-localize in the outer layer of lamina II, as can be observed by the yellow colour in the overlay image, while no co-localization exists with IB4 positive fibers in the inner sublayer of lamina II. (b-b´´´) Triple staining of FRMD6 (b´), SP (b´´) and I-B4 (b´´´) in xenopus spinal cord. Co-localization of FRMD6 with SP at the entrance of the dorsal root in the spinal cord is evident by the yellow colour in the overlay image, while I-B4 positive fibres represent a separate fiber population. (c-c´´) Individual fluorescence channels of Fig. 4a in the main text. Scale bar 100 µm (a-b´´´) 50 µm (c-c´´)

**Figure S5** FRMD6-ir nerve fibers in xenopus lung tissues colocalize with SP. Double immunostaining of FRMD6 (a´, b´, c´) with SP (a´´, b´´, c´´) and overlay images (a, b, c). (a-a´´) Spindle-shaped, contorted terminal endings of large calibre fibers in the region of the lung hilus extensively co-localize with SP. (b-b´´) Image shows a section through the lung wall with the alveolar epthelium (AE) below and a pulmonary blood vessel (BV). In close association to the blood vessel wall large calibre FRMD6/SP-ir fibers can be observed. The arrow points to a small calibre FRMD6/SP-ir fiber in the submucosa beneath the alveolar epithelium. (c-c´´) Arrows point to FRMD6/SP-ir fibers running parallel to the strands of smooth musculature (sm) in the pulmonary walls. Scale bar 100 µm

**Figure S6** The image represents a frontal section through the entire right and left telecephalic hemispheres of an adult xenopus brain at the level immediately posterior to the entry of the accessory olfactory bulb. FRMD6-ir is shown in green, HuC in red. A dense FRMD6-ir fiber plexus highlights the striatum and the accumbens nucleus. Additionally, individual fibers can be observed coursing in the lateral septal area and to a lesser extent in the medial pallium. The image was recorded by the NanoZoomer from the company Hamamatsu using the fluorescence option. Abbr.: ventricle (vl), dorsal pallium (DP), lateral pallium (LP), lateral septum (LS), striatum (STR), nucleus accumbens (Nc), Scale bar 500 µm

**Figure S7 a-d´´** FRMD6-ir fibers in the rat diencephalon. (a-b´´) Composite and individual fluorescence channels of Fig. 5 in the main text. (c-c´´) Image shows the suprachiasmatic nucleus (SCh) overlying the optic chiasm (ox). Third ventricle (III). FRMD6-ir fibers can be observed in the SCh, which is highlighted by numerous AVP-ir fibers, but both markers occur in separate fiber populations. (d-d´´) AVP positive neurons in the supraoptic magnocellular nucleus (SO) lateral to the optic chiasm (ox). FRMD6-ir fibers are visible (d´), but no co-localization with AVP-ir fibers (d´´) occurs. Scale bar 100 µm

**Figure S7 e,f** Triple immunostaining for FRMD6, AVP and SP in the diagonal band of Broca and the septum. (e-e´´´) Vertical limb of the diagonal band of Broca. Composite image (e) and the individual three fluorescence channels for FRMD6 (e´), AVP (e´´) and SP (e´´´) immunostaining are displayed. For the assessment of co-localization in nerve fibers, the overlay images of the green and red channel (e´+ e´´) and the green and magenta channel (e´+ e´´´) are additionally shown. FRMD6-ir fibers co-localize extensively with either AVP-ir or SP-ir fibers, as can be observed by the yellow colour in (e´+e´´) representing FRMD6/AVP double positive fibers, or white colour in (e´+e´´´) indicating FRMD6/SP double positive fibers. Arrows point to two FRMD6-ir fibers, which are negative for both AVP and SP. (f-f´´´) Intermediate part of the lateral septal nucleus (LSI) medial to the lateral ventricle (LV). Composite image of all three fluorescence channels (f), the individual three channels for FRMD6 (f´), AVP (f´´) and SP (f´´´), the overlay image of the green and red channel (f´+f´´) and the overlay image of the green and magenta channel (f´+f´´´). FRMD6-ir fibers co-localize with either AVP, as can be seen by the yellow colour in (f´+ f´´), or with SP, visible by the white colour in (f´+f´´´). Arrow points to a FRMD6 positive nerve fiber, which is negative for both AVP and SP and, therefore, represents a third subpopulation of FRMD6-ir fibers. Scale bar 50µm

**Figure S7 g-j´´** FRMD6-ir fibers in the rat diencephalon. (g-g´´) FRMD6-ir fiber plexus in the lateral habenular nucleus (LHb), lateral to the medial habenular nucleus (MHb) and the dorsal part of the third ventricle (D3V). A significant subpopulation of FRMD6-ir fibers (g´) shows co-localization with SP (g´´), as can be observed by the yellow coloured fibers in the composite image (g). (h-h´´) Two confocal z-series stained for FRMD6 (h´) and AVP (h´´) are fused to a single image covering the area of the lateral habenular nucleus (LHb) and the stria medullaris of the thalamus (sm). AVP-ir fibers show significant co-localization with FRMD6-ir fibers in the part of the LHb abutting the MHb and in nerve fibers of the sm. Hippocampal cortex (CA). (i-i´´) In the adenohypophysis a single FRMD6-ir fiber (i´) double positive for SP (i´´) can be ovserved. (j-j´´) In contast, FRMD6-ir fibers in the neurohypophysis (j´) represent a separate fiber population from SP-ir fibers (j´´) and no co-localization can be seen in the overlay image (j). Scale bar 100 µm

**Figure S8** **a-b´´´** Composite and individual fluorescence channels of Fig. 6 in the main text. In the fluorescence channel for SP immunofluorescence (b´´´) more vesicles are visible compared to the red channel (b´´), which shows the autofluorescence of the PPTA-FusionRed fusion protein. However, secretory granules are acidic organelles, which increase their volume and acidity upon secretion (Pothos et al. 2002). With increasing acidity the autofluorescence of the fusion protein decreases due to acid quenching, which explains the mismatch between the number of secretory granules detected by the autofluorescence moiety of the FusionRed protein and SP immunofluorescence. (c-d´´´) Empty vector control for MCF-7 cells (c-c´´´) and AtT-20 cells (d-d´´´). The empty FusionRed vector without the PPTA protein moiety was expressed and an immunofluorescence analysis under identical conditions as in (a-a´´´) and (b-b´´´) was performed. The FusionRed autofluorescent protein is localized diffusely in the cytoplasm and in the cell nucleus (c´´, d´´). No unspecific crossreaction of the FRMD6 antiserum (c´, d´) with the FusionRed protein can be observed, nor any crossreaction of the SP immunofluorescence. Scale bar 10 µm

**References cited in supplementary text**

Cubois-Dauphin M, Zakarian S (1987) Distribution of the C-terminal glycopeptide of the vasopressin prohormone in rat brain: an immunocytochemical study. Neuroscience 21: 903-921

Pothos EN, Mosharov E, Liu KP, Setlik W, Haburcak M, Baldini G, Gershon MD, Tamir H, Sulzer D (2002) Stimulation-dependent regulation of the pH, volume and quantal size of bovine and rodent secretory vesicles. J Physiol 542: 453-476
